# Supplementary material for: MTCH2 promotes BAX and BAK self-assembly and apoptotic pore growth
Source: Nat Struct Mol Biol. 2026 Apr 29;33(5):824–37. doi: 10.1038/s41594-026-01805-8 (PMC13186707; doi:10.1038/s41594-026-01805-8)
Supplement: Supplementary file 1 — Supplementary Figs. 1–6 and Methods. [file 41594_2026_1805_MOESM1_ESM.pdf]

nature structural & molecular biology

Supplementary information https://doi.org/10.1038/s41594-026-01805-8

MTCH2 promotes BAX and BAK self-assembly and apoptotic pore growth

In the format provided by the authors and unedited

Supplementary information

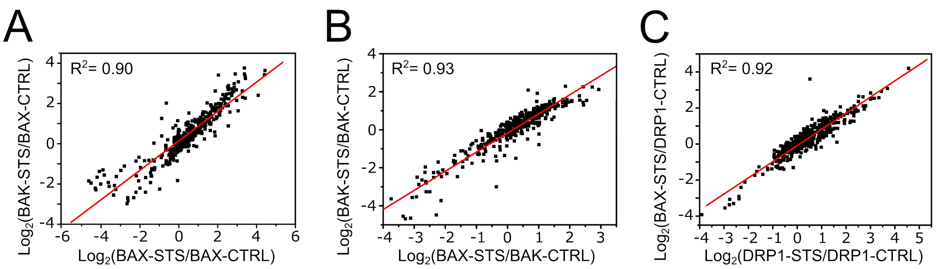

Figure S1. Analysis of APEX2 experiments.

A, B, C) Scatter plots of Log_2 (STS/CTRL) values for APEX2-BAX (A), APEX2-BAK (B) and APEX2-DRP1 (C). R2 of each linear regression is indicated.

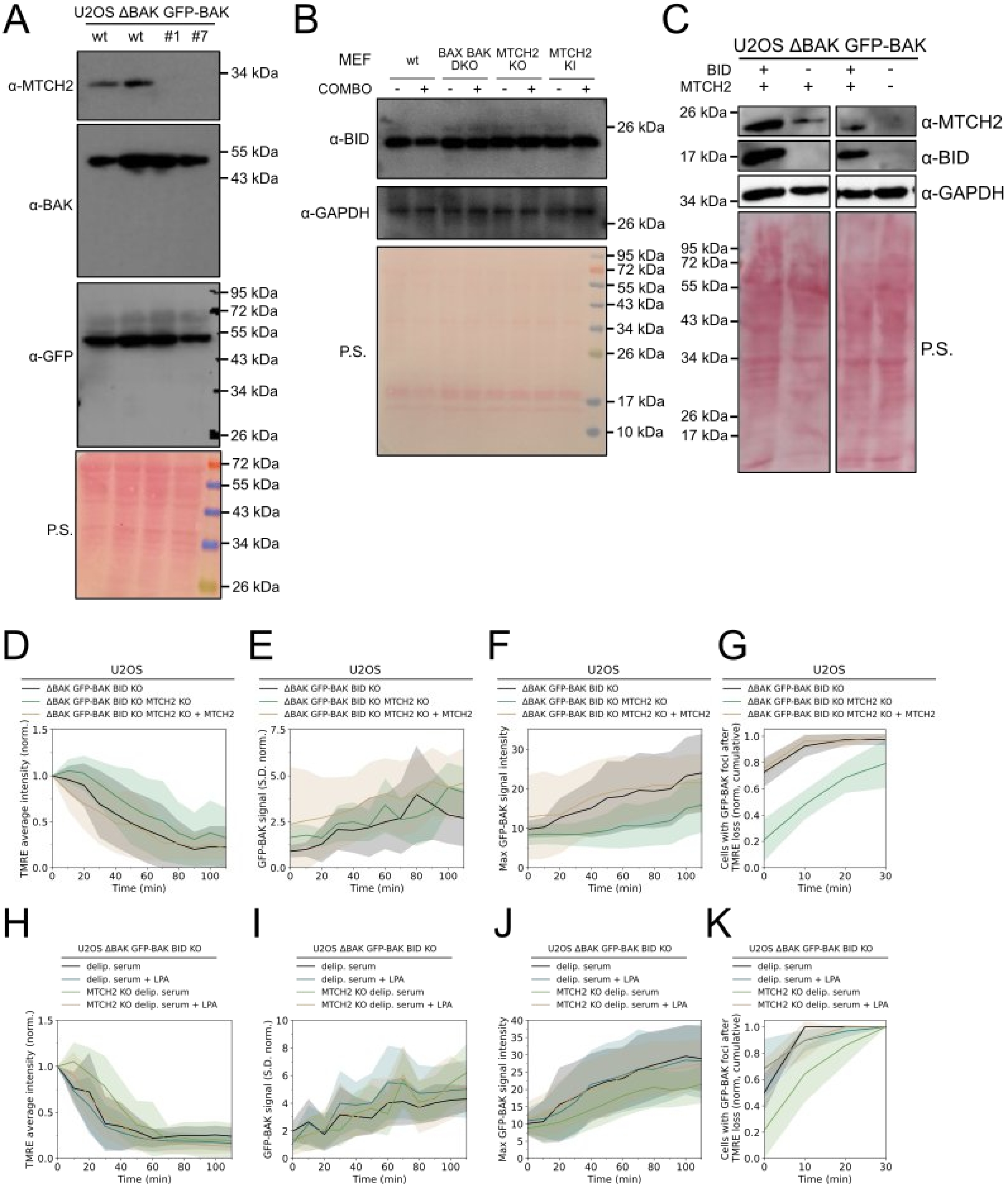

Figure S2. Role of MTCH2 and BID on BAK high order assembly.

A) Representative WB of MTCH2 expression levels in U2OS ΔBAK cells expressing GFP-BAK and MTCH2 KO U2OS ΔBAK cells expressing GFP-BAK, clones #1 and #7

B) Representative WB of BID expression levels in wt, BAX/BAK DKO, MTCH2 KO and MTCH2 KI MEFs, with (+) or without (-) apoptosis induction with 10 μM COMBO and 20 μM QVD for 3 h.

C) Representative WB of MTCH2 and BID expression levels in BID KO U2OS ΔBAK cells expressing GFP-BAK and BID KO MTCH2 KO U2OS ΔBAK cells expressing GFP-BAK.

D-G) Quantification of mitochondrial potential loss and GFP-BAK oligomerization in BID KO U2OS ΔBAK cells expressing GFP-BAK and in BID KO MTCH2 KO U2OS ΔBAK cells expressing GFP-BAK and reconstituted or not with MTCH2. D) Average TMRE signal over time normalized to each cell type. E, F) Accumulation of BAK into discrete foci quantified as S.D. of the GFP signal (E) or GFP maximum signal (F) over time. Values normalized to wt cells. G) Cumulative quantification of the number of cells presenting GFP-BAK foci at different times after TMRE loss. More than 20 cells from n=3 independent experiments were analyzed. Solid lines connect mean values at different time points. Colored areas correspond to S.D.

H-K) Quantification of mitochondrial potential loss and GFP-BAK oligomerization in BID KO U2OS ΔBAK cells expressing GFP-BAK and BID KO MTCH2 KO U2OS ΔBAK cells expressing GFP-BAK incubated in media with delipidated serum, in presence or absence of 20 µM lysophosphatidic acid (LPA) for 18 h, prior to microscopic analysis. Analysis was performed similar to (D-G). More than 20 cells from n=3 independent experiments were analyzed. Solid lines connect mean values at different time points. Colored areas correspond to S.D.

A-C) P.S.: Ponceau staining.

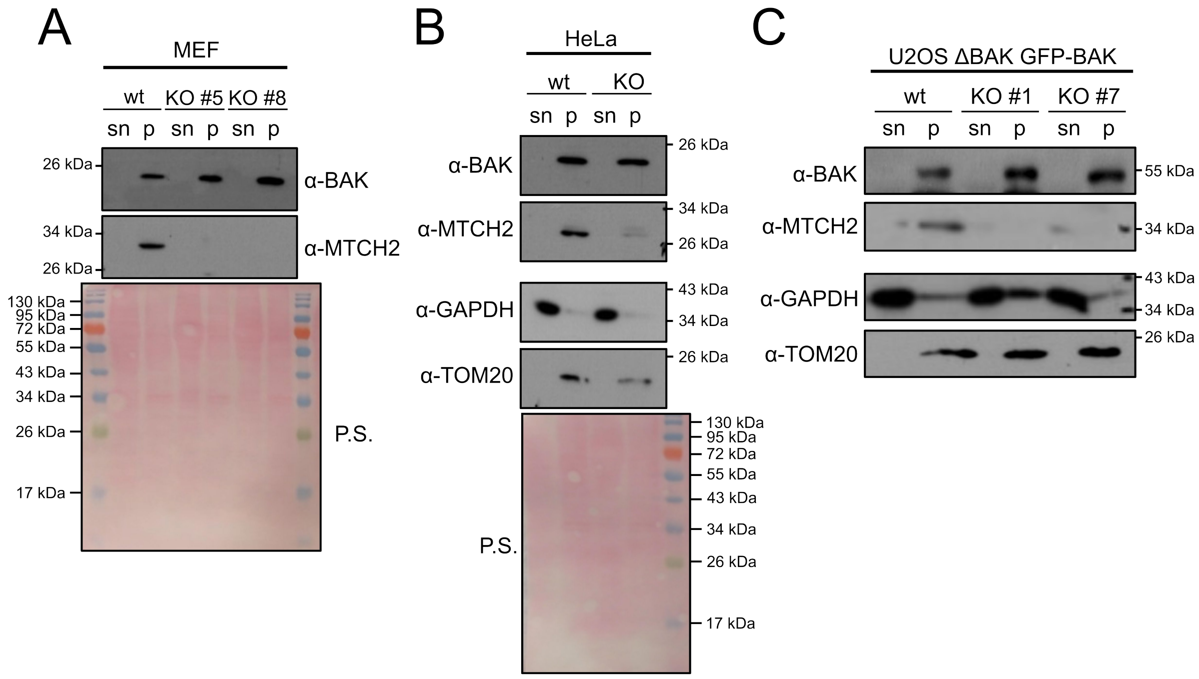

Figure S3. Mitochondrial targeting of BAK in MTCH2 KO cells.

A-C) Subcellular localization of BAK and GFP-BAK, in WT and MTCH2 KO MEF cells (A), HeLa cells (B) and (C) U2OS ΔBAK cells expressing GFP-BAK. P.S.: Ponceau staining.

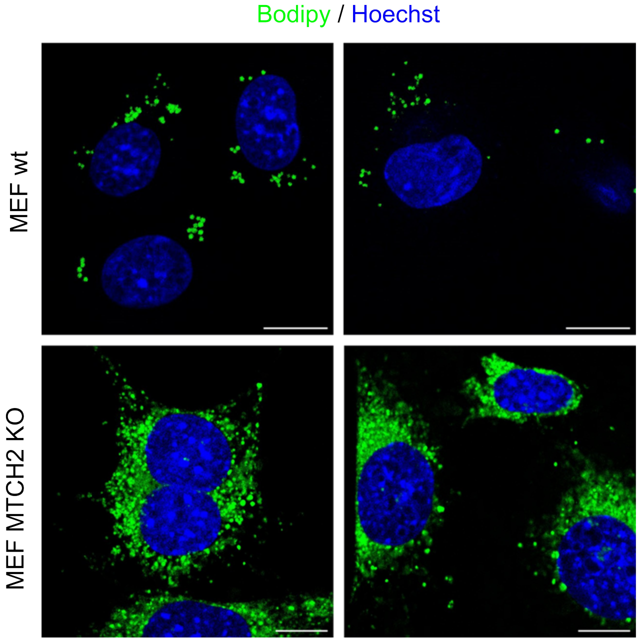

Figure S4. Representative images of lipid droplets staining in MEF WT and MTCH2 KO cells.

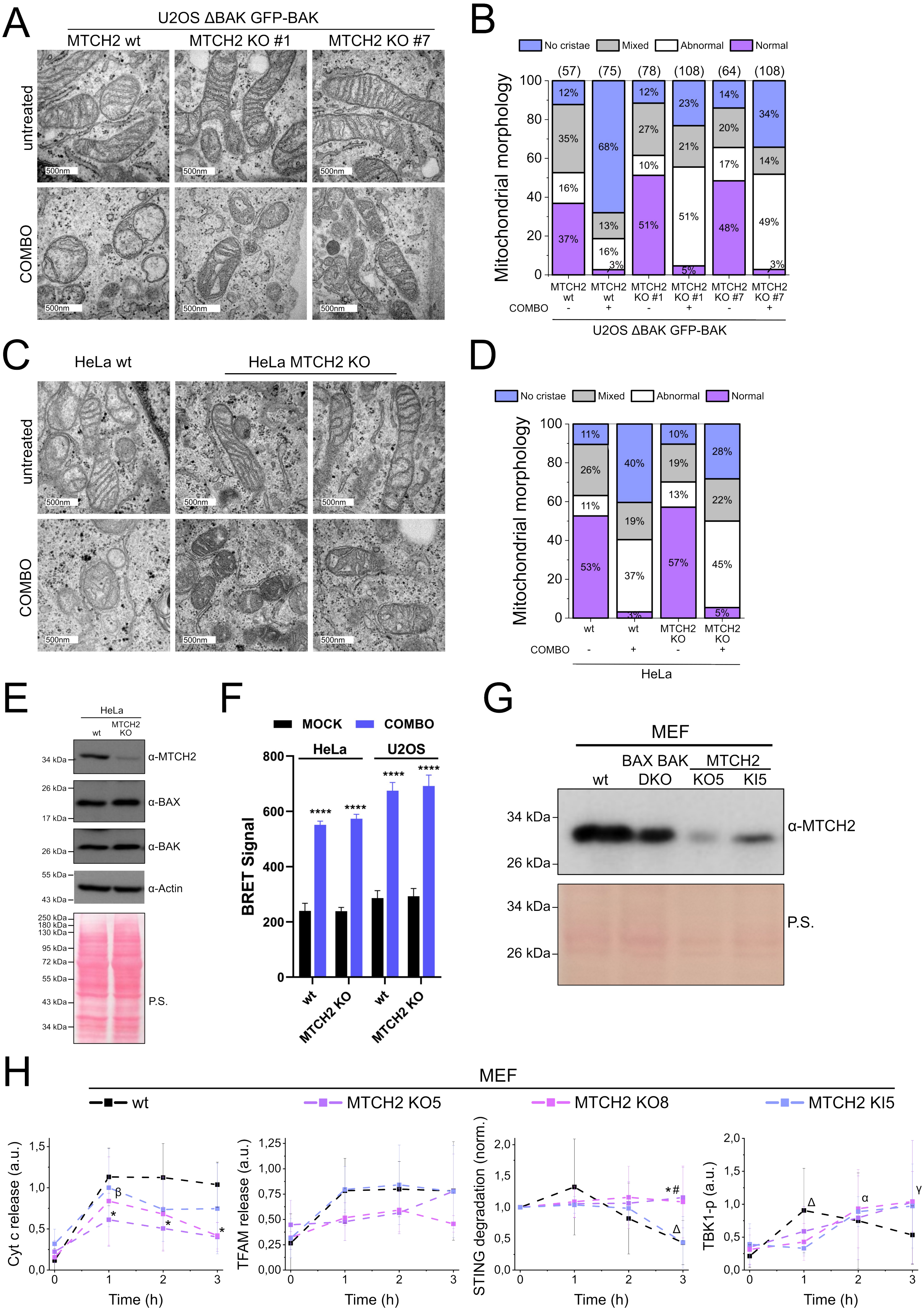

Figure S5. Analysis of mitochondrial organization in MTCH2 deficient cells.

A,C) Representative EM images of mitochondrial cristae from U2OS ΔBAK cells expressing GFP-BAK and lacking or not MTCH2 (A), and wt and MTCH2 KO HeLa cells (B) treated with 5 μM COMBO and 20 μM QVD for 3 h. Scale bar, 500 nm.

B,D) Quantification of A and C with classification into: 1) no cristae, 2) abnormal cristae (swollen, abnormal distribution), 3) normal (classical elongated cristae with uniform distribution) and 4) mixed phenotype. n=4-6 independent cells. In brackets appears the total amount of mitochondria considered.

E) Representative WB of MTCH2 expression levels in wt and bulk MTCH2 KO HeLa cells. P.S.: Ponceau staining.

F) BRET signal correlated with mitochondria-ER distances in HeLa and U2OS cells untreated and upon apoptosis induction 1 μM COMBO, normalized to the wt untreated value. Data from 9 technical replicates from n=3 independent experiments. Error is S.D. *** p<0,001, unpaired student t test.

G) Representative WB of MTCH2 expression levels in wt, BAX BAK DKO and bulk MTCH2 KO and KI MEF cells. P.S.: Ponceau staining.

H) Quantification of the WBs shown in Figure 6F,G, from n=4-7 independent experiments. Mean values +/- S.D. β =p<0,05 comparing wt and KI at the selected time point. *=p<0,05 comparing wt and KO #5 at the selected time point. # =p<0,05 comparing wt and KO #8 at the selected time point. Δ, γ and α =p<0,05 comparing wt to wt, KO and KI respectively, at the selected time point. Unpaired Student t test.

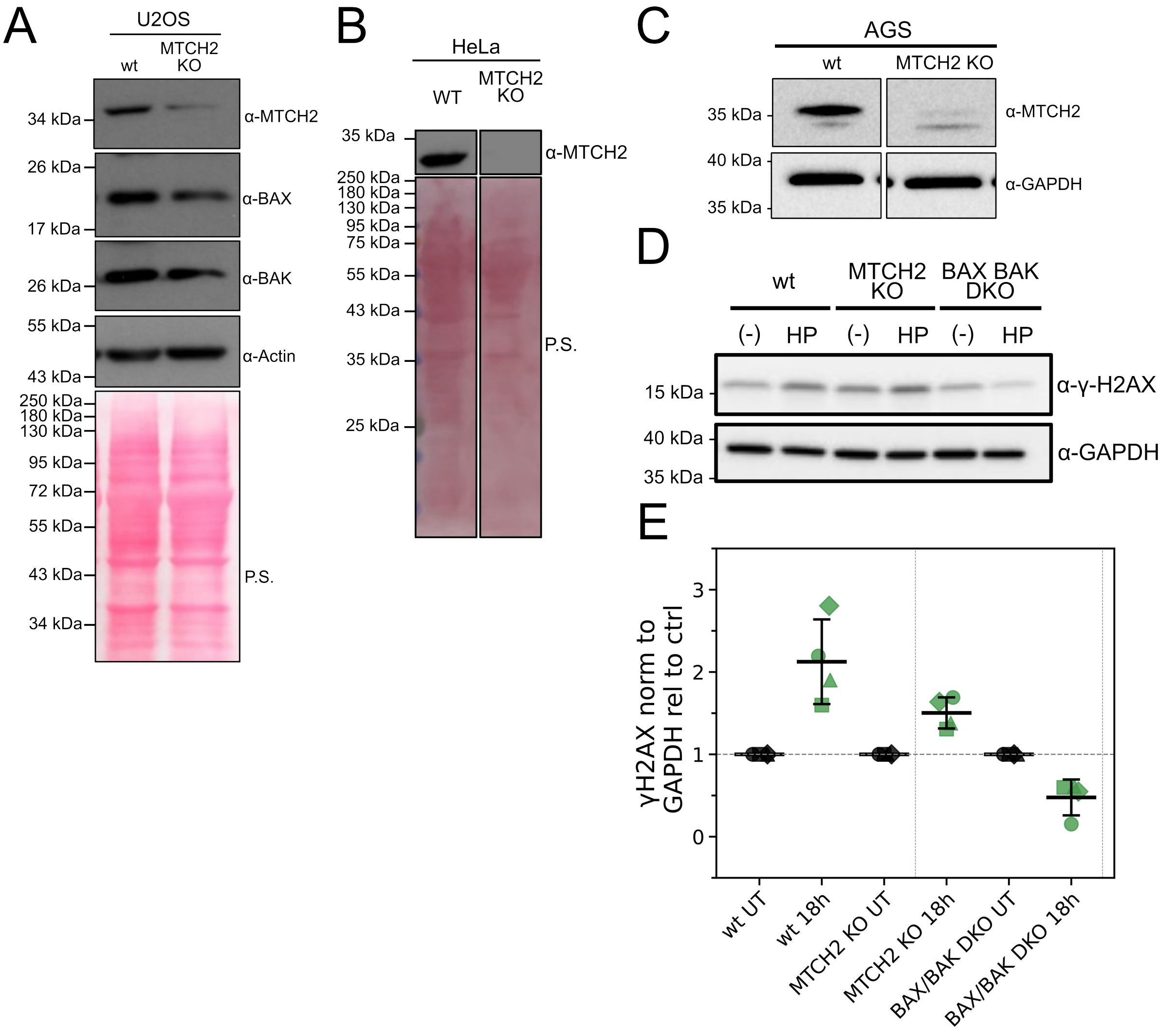

Figure S6. Role of MTCH2 on cell death sensitivity and on sublethal MOMP upon bacterial infection.

A-C) Representative WB of MTCH2 expression levels in wt and MTCH2 KO U2OS (A), HeLa cells (B) and (C) AGS cells. P.S.: Ponceau staining, acting and/or GADPH as loading controls.

D) Representative WB of H2Aγ in cellular extracts of wt, BAX/BAK DKO and MTCH2 KO HeLa cells infected or not (-) with *Helicobacter pylori* (*HP*). Loading control, GAPDH. n=4 independent experiments.

E) Quantification of data in D. γH2AX levels upon *Helicobacter pylori* infection in the indicated samples were normalized to the total protein concentration and to the control of each specific cell line. n=4 independent experiments.

Extended Materials and Methods

MERLIN assay for mitochondria-ER contacts

To assess the distance between the endoplasmic reticulum and the mitochondria in HeLa and U2OS cells we used a BRET-based biosensor called MERLIN as described in [47,48]. Briefly, wt and MTCH2 KO HeLa and U2OS cells were seeded onto a white 96 well white-plate for 24 h and transfected with Rluc-L1-B33C only (donor in the BRET couple) or with MERLIN (donor and Scal-L1-mVenus, acceptor) and induced apoptosis with 1 µM COMBO (ABT-737 and S63845) including 10 µM QVDA for 3 h in phenol red free media. Next, cells were incubated with 5 µM Coelenterazine h (Promega) for 5 min in the dark. BRET measurements were carried out at RT in a Paradigm detection platform (Beckman courtier) with a BRET1 cartridge. BRET signal was calculated as acceptor emission relative to donor emission and corrected by subtracting the background ratio value detected when Rluc8 is expressed alone and normalized to the untreated wt conditions.

Lipid droplets analysis.

Wt and MTCH2 KO MEF cells were incubated with 1 µM BODIPY 493/503 (D-3922; Life Technologies) and 1µg/ml Hoechst in complete media for 20 min at 37 °C, washed with PBS and fixed with 4% PFA. Confocal microscopy was performed on an TCS SP8 gSTED 3X, Leica Microsystems inverted microscope equipped with a Plan-Apochromat 63x/1.4 oil immersion objective and a White Light Laser (WLL). Pinhole size was adjusted to 1 Airy unit.

Supplementary Figures Source Data western blots

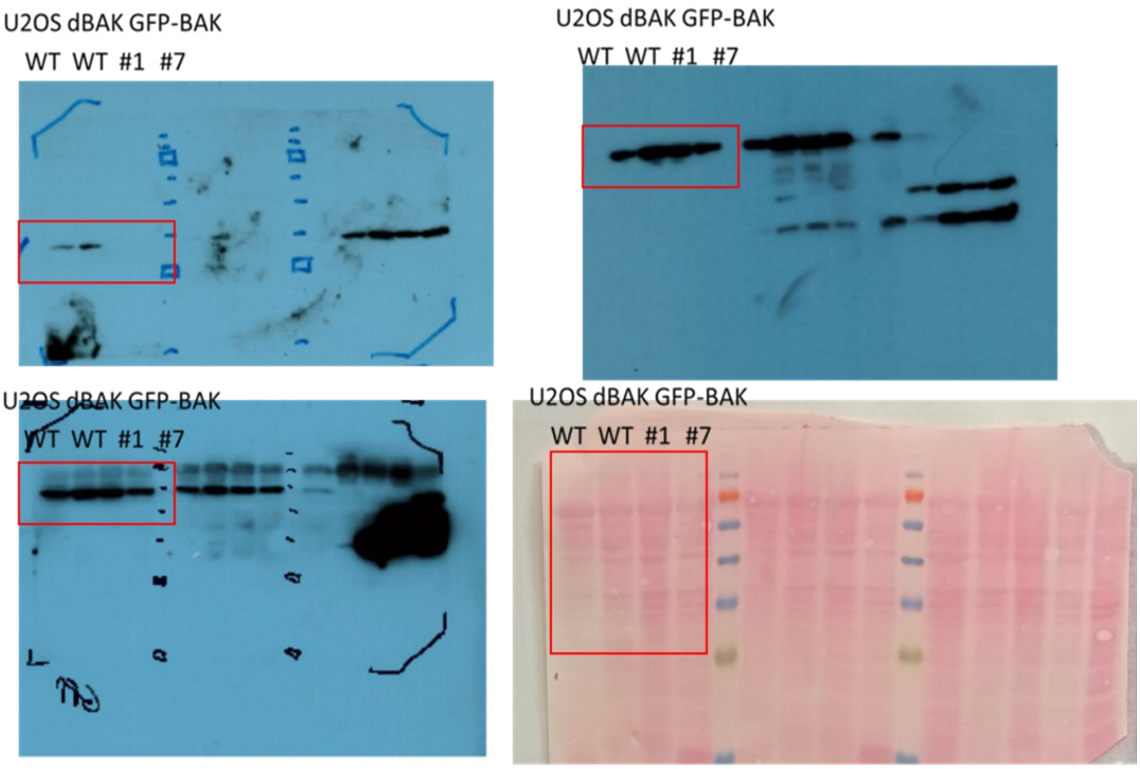
U2OS dBAK GFP-BAK
WT WT #1 #7
U2OS dBAK GFP-BAK
WT WT #1 #7
U2OS dBAK GFP-BAK
WT WT #1 #7
U2OS dBAK GFP-BAK
WT WT #1 #7

Figure S2A

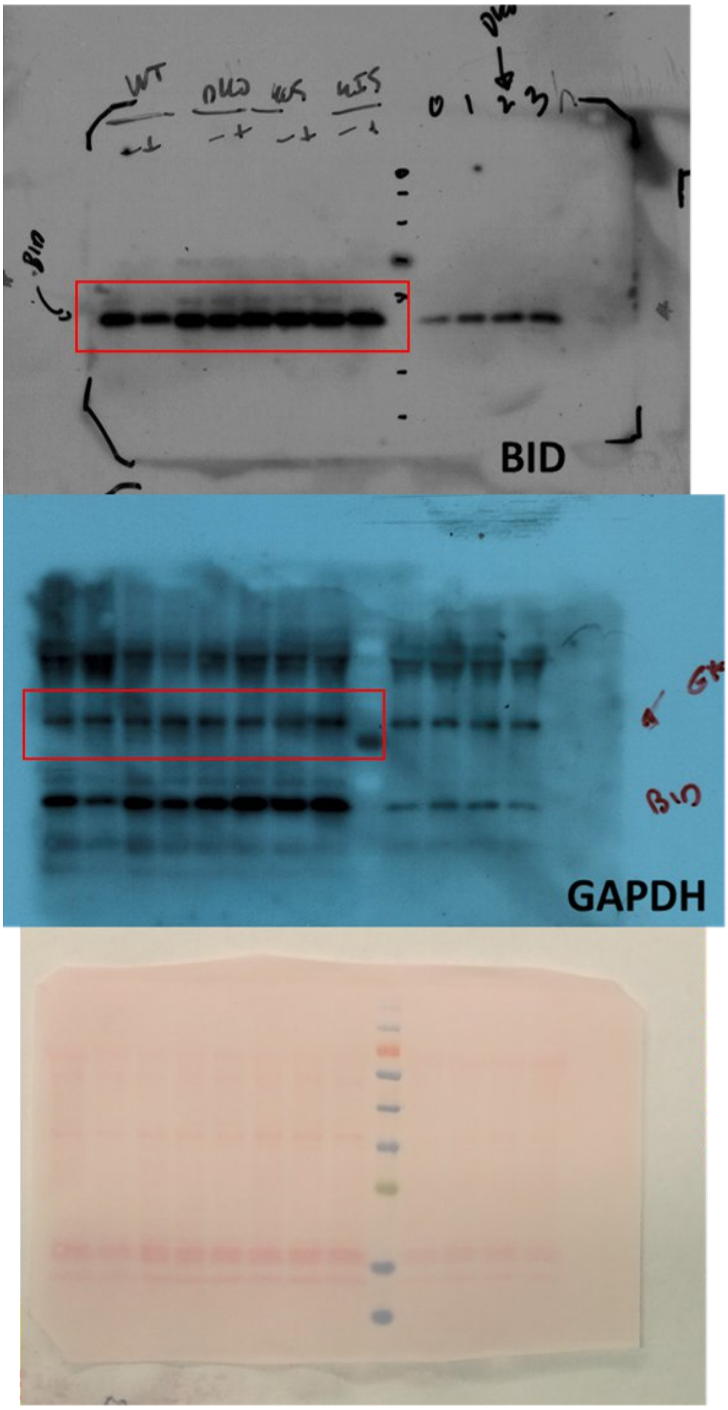

Figure S2B

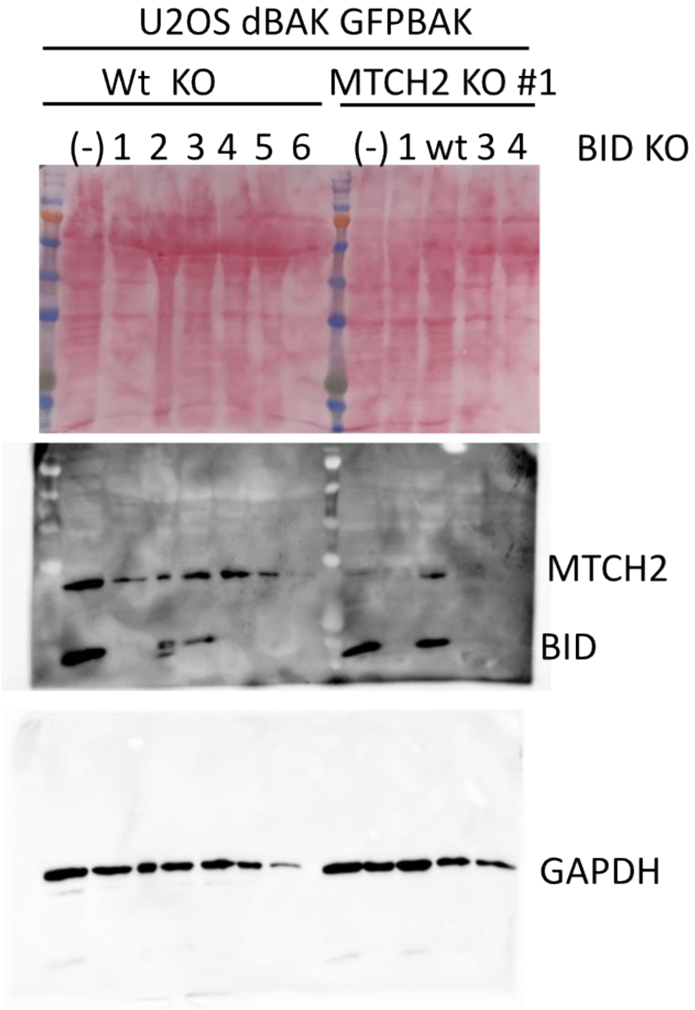
U2OS dBAK GFPBAK
Wt KO
MTCH2 KO #1
(-) 1 2 3 4 5 6 (-) 1 wt 3 4
BID KO
MTCH2
BID
GAPDH

Figure S2C

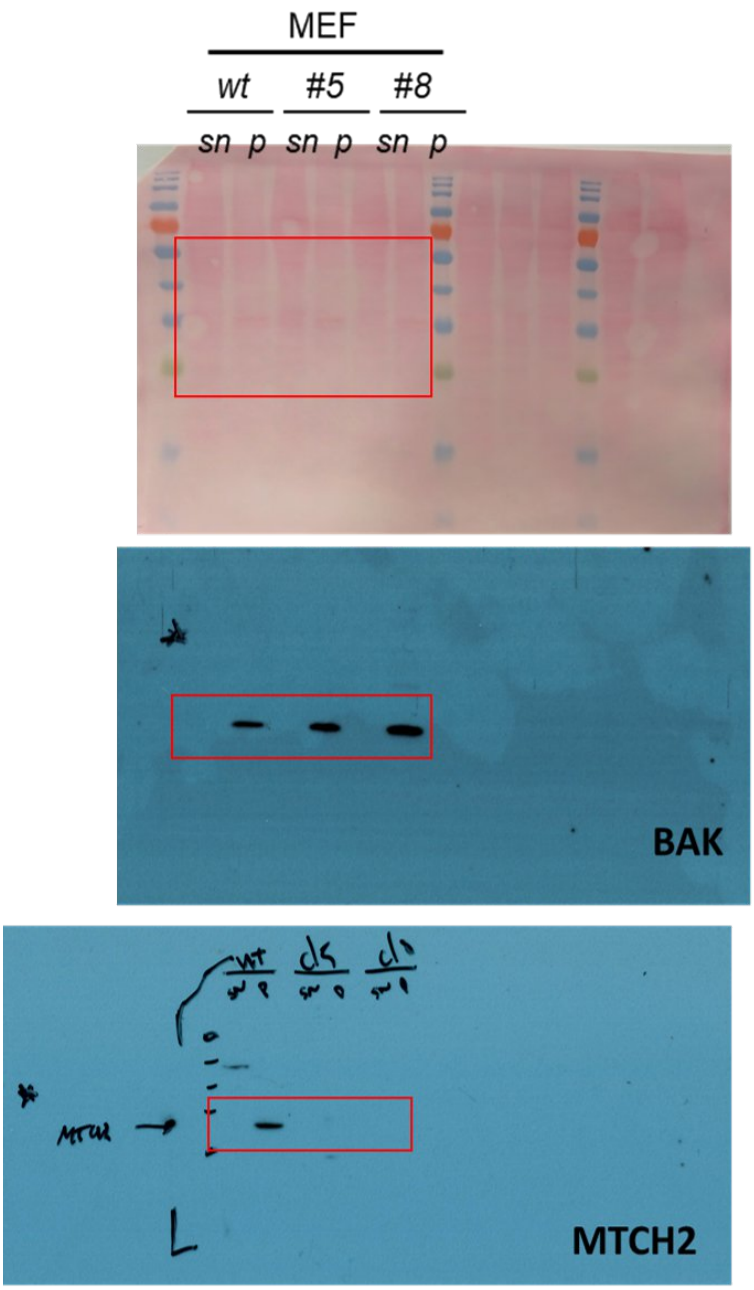

Figure S3A

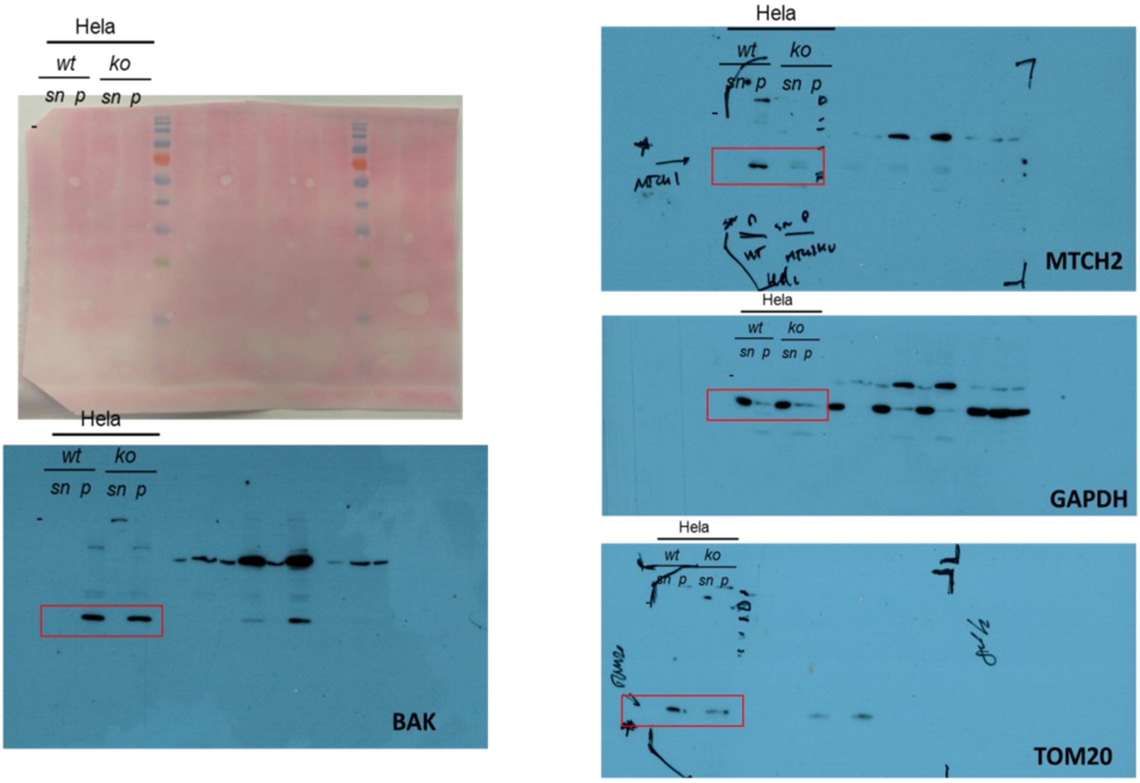

Figure S3B

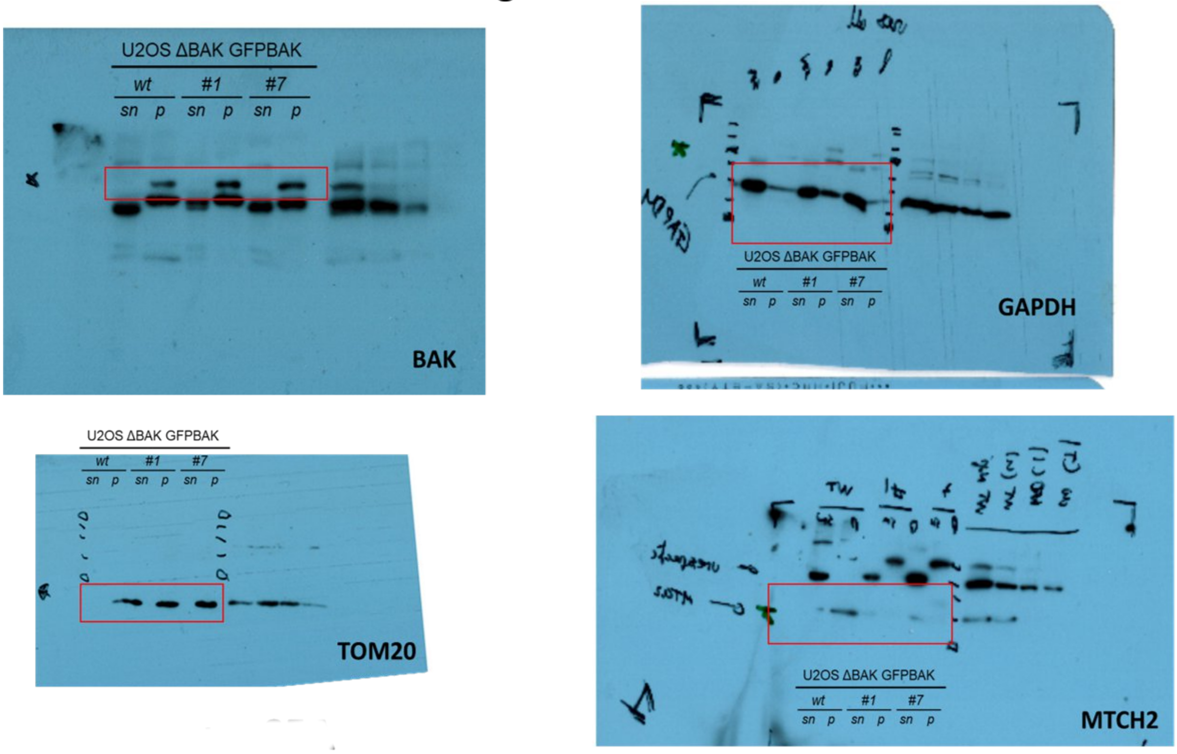

Figure S3C

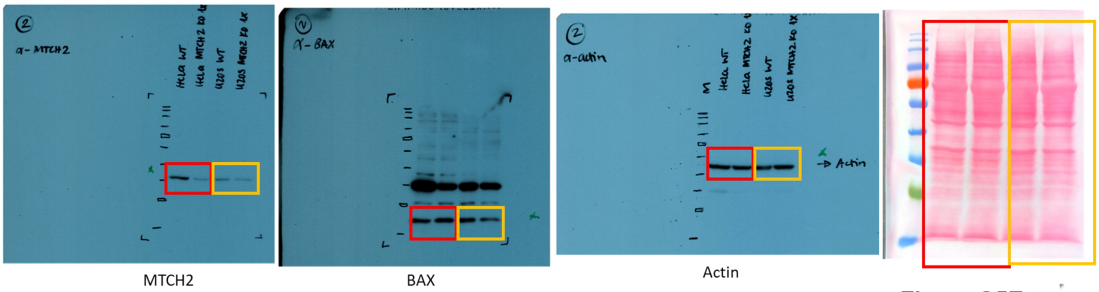

MTCH2 BAX Actin

Figure S5E

MEFwt DKO KO5 KI5

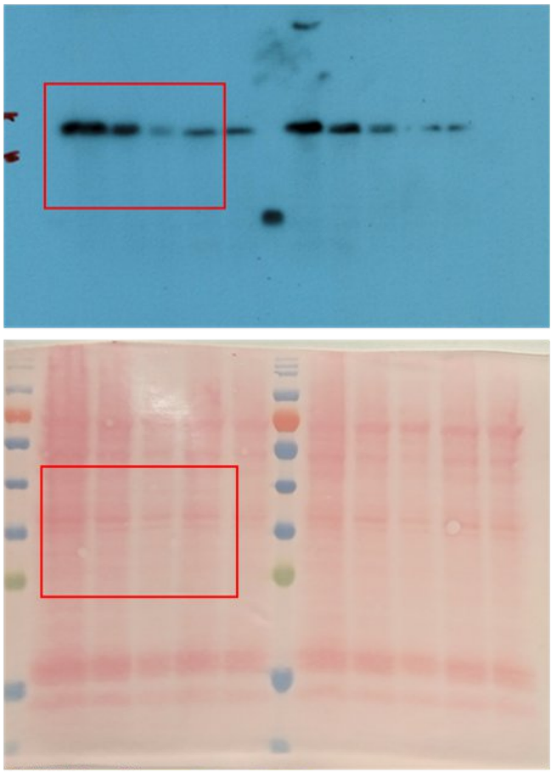

Figure S5G

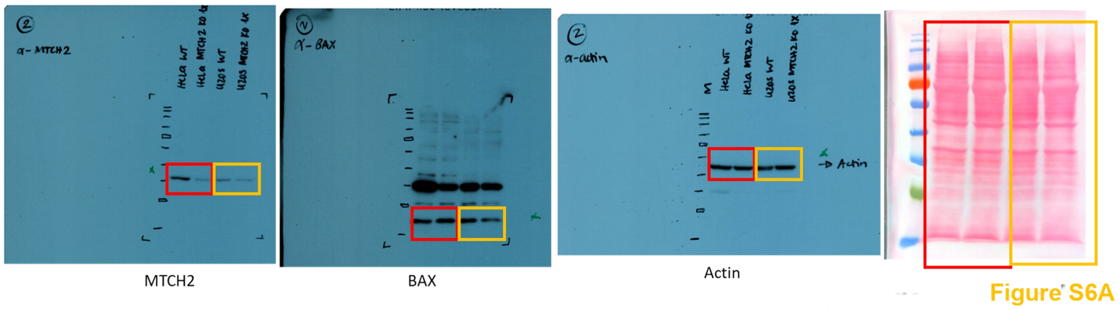

MTCH2 BAX Actin

Figure S6A

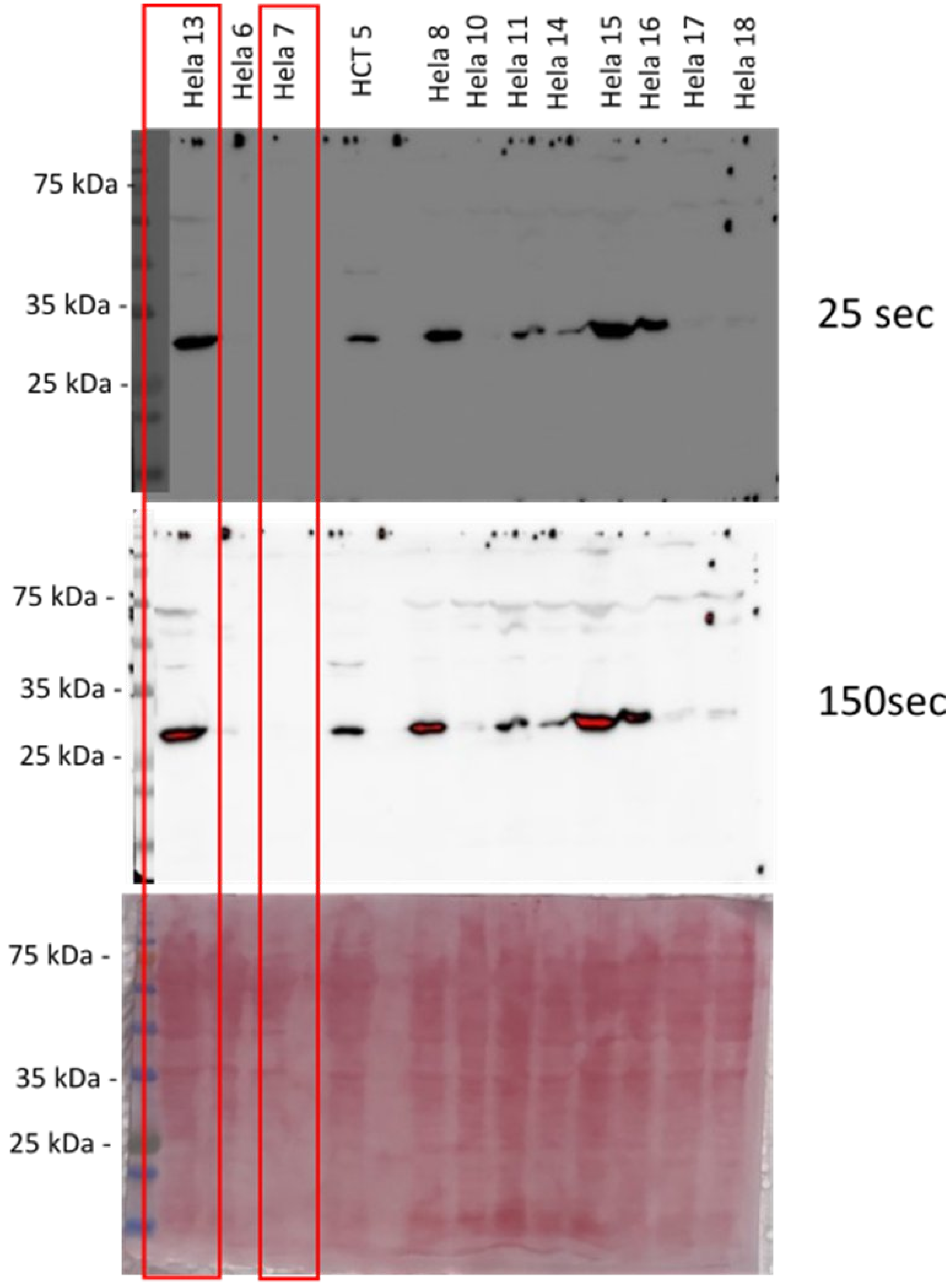
Hela 13
Hela 6
Hela 7
HCT 5
Hela 8
Hela 10
Hela 11
Hela 14
Hela 15
Hela 16
Hela 17
Hela 18
75 kDa -
35 kDa -
25 kDa -
25 sec
75 kDa -
35 kDa -
25 kDa -
150sec
75 kDa -
35 kDa -
25 kDa -

Figure S6B

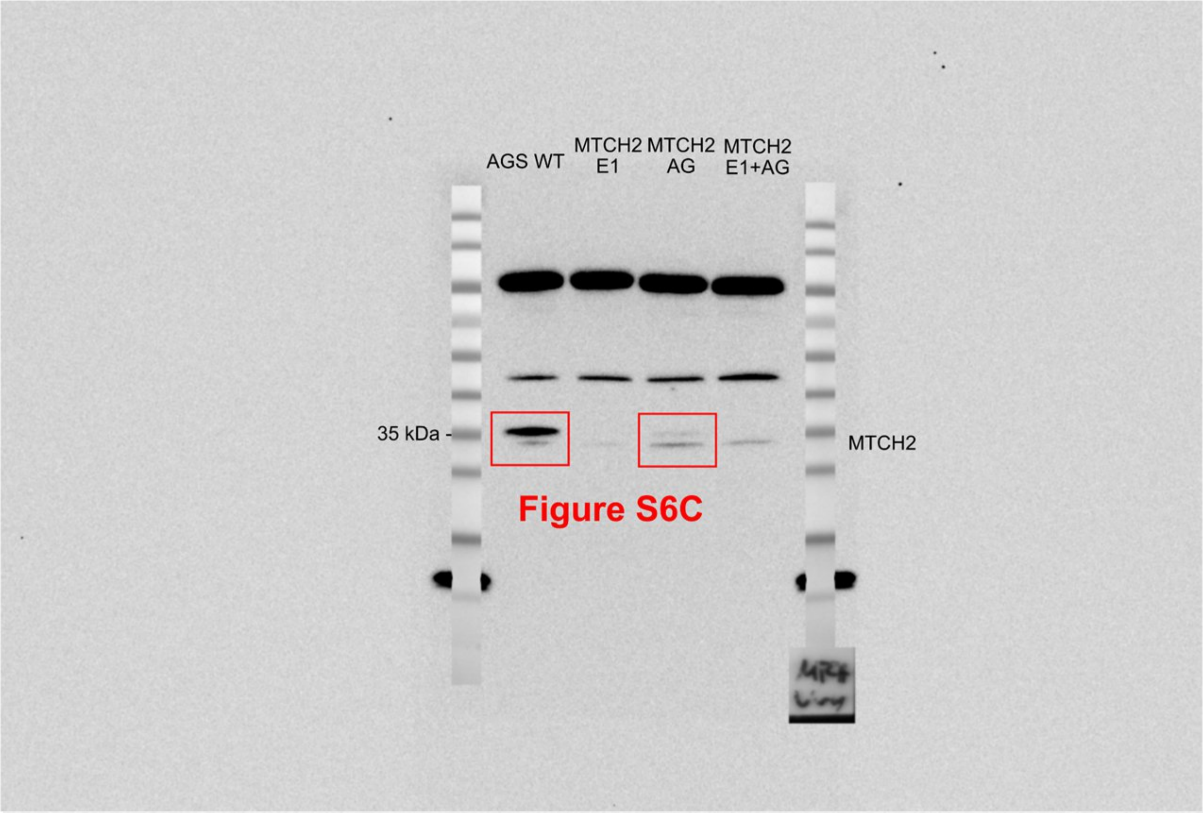
AGS WT
MTCH2 E1
MTCH2 AG
MTCH2 E1+AG
35 kDa
MTCH2
Figure S6C

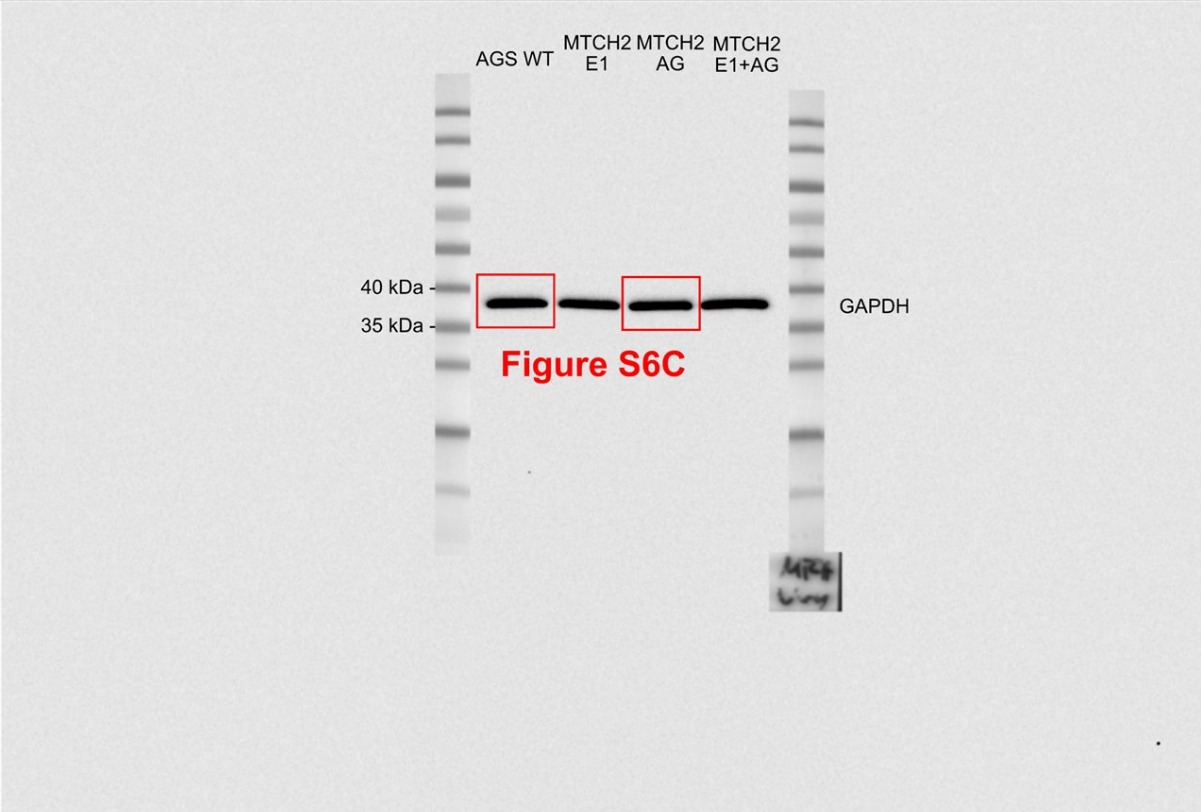
AGS WT
MTCH2 E1
MTCH2 AG
MTCH2 E1+AG
40 kDa
35 kDa
GAPDH
Figure S6C

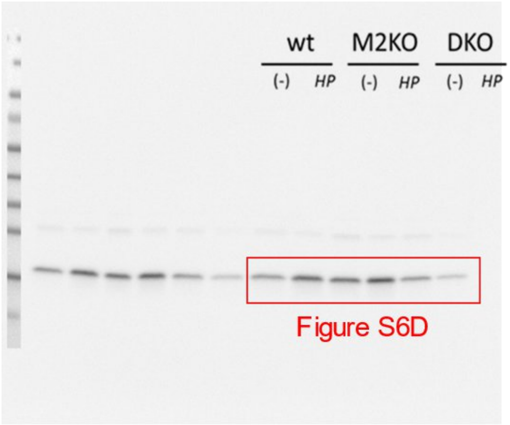

G-H2Ax

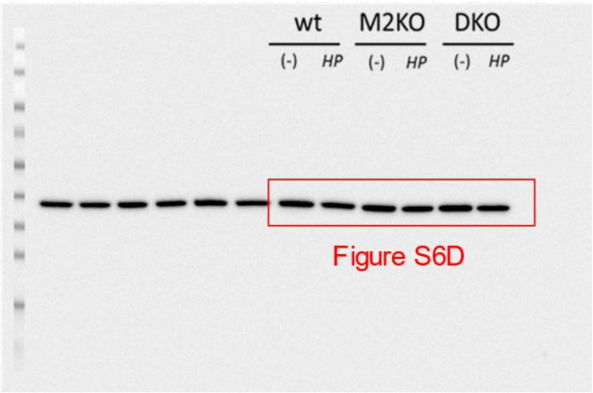

GAPDH
