# Supplementary material for: MTCH2 promotes BAX and BAK self-assembly and apoptotic pore growth
Source: Nat Struct Mol Biol. 2026 Apr 29;33(5):824–37. doi: 10.1038/s41594-026-01805-8 (PMC13186707; doi:10.1038/s41594-026-01805-8)
Supplement: Supplementary file 2 — Reporting Summary [file 41594_2026_1805_MOESM2_ESM.pdf]

Corresponding author(s): Ana J Garcia-Saez

Last updated by author(s): 2025/12/23

Reporting Summary

Nature Portfolio wishes to improve the reproducibility of the work that we publish. This form provides structure for consistency and transparency in reporting. For further information on Nature Portfolio policies, see our Editorial Policies and the Editorial Policy Checklist.

Statistics

For all statistical analyses, confirm that the following items are present in the figure legend, table legend, main text, or Methods section.

n/a	Confirmed	
☐	☒	The exact sample size (*n*) for each experimental group/condition, given as a discrete number and unit of measurement
☐	☒	A statement on whether measurements were taken from distinct samples or whether the same sample was measured repeatedly
☐	☒	The statistical test(s) used AND whether they are one- or two-sided<br>*Only common tests should be described solely by name; describe more complex techniques in the Methods section.*
☒	☐	A description of all covariates tested
☐	☒	A description of any assumptions or corrections, such as tests of normality and adjustment for multiple comparisons
☐	☒	A full description of the statistical parameters including central tendency (e.g. means) or other basic estimates (e.g. regression coefficient) AND variation (e.g. standard deviation) or associated estimates of uncertainty (e.g. confidence intervals)
☐	☒	For null hypothesis testing, the test statistic (e.g. *F*, *t*, *r*) with confidence intervals, effect sizes, degrees of freedom and *P* value noted<br>*Give P values as exact values whenever suitable.*
☒	☐	For Bayesian analysis, information on the choice of priors and Markov chain Monte Carlo settings
☒	☐	For hierarchical and complex designs, identification of the appropriate level for tests and full reporting of outcomes
☒	☐	Estimates of effect sizes (e.g. Cohen's *d*, Pearson's *r*), indicating how they were calculated

Our web collection on statistics for biologists contains articles on many of the points above.

Software and code

Policy information about availability of computer code

Data collection	For proteomic assays, peptide mixtures were analyzed on an Easy-nLC II coupled to an LTQ Orbitrap XL mass spectrometer.<br>Lipidomic data were acquired using Analyst 1.7.3 software (SCIEX).<br>For microscopy imaging we used Zeiss, Leica and JEOL software.<br>Further details are within the manuscript.
Data analysis	Proteomic analysis: acquired MS spectra were processed with MaxQuant software package version 1.5.2.8 with integrated Andromeda search engine. Database search was performed against a target-decoy Homo sapiens database obtained from Uniprot.(more details in the manuscript). Proteomic data will be available in PRIDE upon publication (Project accession: PXD055201).<br>Lipidomic data:Extraction of lipids from isolated mitochondria and analysis of PC, PE, PI, PS, PG, and PA species by Nano-Electrospray Ionization Tandem Mass Spectrometry were performed as in Dadsena et al 2024 Nature Communications. Lipidomic data are available in Zenodo (https://doi.org/10.5281/zenodo.19006330).<br>Microscopy data was analysed using microscope-specific software and Fiji.<br>Stoichiometry data: To analyze the stoichiometry data in a high throughput manner, a Python script was developed, that is available under https://github.com/jaufdermauer/stoichiometry_analysis (default main_dbd branch). The function of the code is explained in detail in the Materials & Methods section. The license and instructions on how to use together with a test dataset are in the repository.

For manuscripts utilizing custom algorithms or software that are central to the research but not yet described in published literature, software must be made available to editors and reviewers. We strongly encourage code deposition in a community repository (e.g. GitHub). See the Nature Portfolio guidelines for submitting code & software for further information.

Data

Policy information about availability of data

All manuscripts must include a data availability statement. This statement should provide the following information, where applicable:

- Accession codes, unique identifiers, or web links for publicly available datasets
- A description of any restrictions on data availability
- For clinical datasets or third party data, please ensure that the statement adheres to our policy

All data supporting the findings of this manuscript are available from the corresponding author upon reasonable request. Proteomic data will be available in PRIDE upon publication (Project accession: PXD055201). Lipidomic data is available in Zenodo under https://doi.org/10.5281/zenodo.19006330

Research involving human participants, their data, or biological material

Policy information about studies with human participants or human data. See also policy information about sex, gender (identity/presentation), and sexual orientation and race, ethnicity and racism.

Reporting on sex and gender	NA
Reporting on race, ethnicity, or other socially relevant groupings	NA
Population characteristics	NA
Recruitment	NA
Ethics oversight	NA

Note that full information on the approval of the study protocol must also be provided in the manuscript.

Field-specific reporting

Please select the one below that is the best fit for your research. If you are not sure, read the appropriate sections before making your selection.

☒ Life sciences ☐ Behavioural & social sciences ☐ Ecological, evolutionary & environmental sciences

For a reference copy of the document with all sections, see nature.com/documents/nr-reporting-summary-flat.pdf

Life sciences study design

All studies must disclose on these points even when the disclosure is negative.

Sample size	The design of the experimental conditions was based on previous knowledge from similar experiments were done in the field. Also, pilot experiments were carried out that indicated the sample sizes chosen were adequate for a robust phenotype.
Data exclusions	One replicate from the lipidomic assay was excluded due to insufficient total lipid content, as indicated in the manuscript.
Replication	All experiments were performed with at least two or three independent replicates. All figures present the means and standard deviations (SD). All experimental replicates successfully validated the experimental findings.
Randomization	The samples were randomized and allocated in a way that made sense for the flow of the study, considering factors such as time and logical progression, without any preferential treatment.
Blinding	The data analysis was performed without blinding

Reporting for specific materials, systems and methods

We require information from authors about some types of materials, experimental systems and methods used in many studies. Here, indicate whether each material, system or method listed is relevant to your study. If you are not sure if a list item applies to your research, read the appropriate section before selecting a response.

Materials & experimental systems

n/a	Involved in the study	
☐	☒	Antibodies
☐	☒	Eukaryotic cell lines
☒	☐	Palaeontology and archaeology
☐	☒	Animals and other organisms
☒	☐	Clinical data
☒	☐	Dual use research of concern
☒	☐	Plants

Methods

n/a	Involved in the study	
☒	☐	ChIP-seq
☒	☐	Flow cytometry
☒	☐	MRI-based neuroimaging

Antibodies

Antibodies used

The study utilized Rabbit polyclonal anti-BAX (Cat#2772; RRID: AB_10695870; Cell Signaling Technology), Rabbit monoclonal anti-BAX (D2E11) (Cat#5023; RRID: AB_10557411; Cell Signaling Technology), Rabbit polyclonal anti-GAPDH (Cat#ab9485; RRID: AB_307275; Abcam), Goat polyclonal anti-Rabbit IgG-HRP (Cat#111-035-003; RRID: AB_2313567; Jackson Immuno Research), Goat polyclonal anti-Mouse IgG-HRP (Cat#115-035-003; RRID: AB_10015289; Jackson Immuno Research), Goat polyclonal anti-Rabbit (AF633) (Cat#A-21070; RRID: AB_2535731; ThermoFisher), Rabbit monoclonal anti-Tom20 (D8T4N) (Cat#42406; RRID: AB_2687663; Cell Signaling Technology), Mouse anti-Cytc (Cat#556433; RRID: AB_396417; BD Biosciences), Mouse monoclonal anti-GAPDH (D4C6R) (Cat#97166; RRID: AB_2756824; Cell Signaling Technology), Rabbit monoclonal anti-pTBK1/NAK, (Ser172) (D52C2) (Cat#5483; RRID: AB_10693472; Cell Signaling Technology), Rabbit monoclonal anti-TBK1/NAK (D1B4) (Cat#3504; RRID: AB_2255663; Cell Signaling Technology), Rabbit monoclonal anti-STING (D2P2F) (Cat#13647; RRID: AB_2732796; Cell Signaling Technology), Mouse monoclonal anti-GFP (Cat#MA5-15256; RRID: AB_10979281; ThermoFisher), anti-mouse IG-HRP (Cat#115-035-166; RRID: AB_2338511; Dianova), anti-rabbit-HRP (Cat#A6667; RRID: AB_258307; Sigma), anti-GAPDH (Cat#MAB374; RRID: AB_2107445; Cell Signaling Technology), anti-MTCH2 (Cat#PA5-88873; RRID: AB_2805186; Thermo Fisher), anti-Phospho-Histone H2A.X (Cat#2577; RRID: AB_2118010; Cell Signaling Technology), anti-SMAC (Cat#15108; RRID: AB_721554; Cell Signaling Technology), anti-AF488-anti mouse (Cat#A-11008; RRID: AB_143165; ThermoFisher), anti-Bak (Cat#12105; RRID: AB_2716685; Cell Signaling Technology), anti-Dlp1 (Cat#611112; RRID: AB_398423; BD Biosciences), anti-Flag (Cat#F3165; RRID: AB_259529; Sigma), anti-Mtch2 (Cat#ab105527; RRID: AB_10866682; Abcam), anti-Streptavidin-AF555 (Cat#S32355; RRID: AB_2571525; ThermoFisher), anti-Streptavidin-HRP (Cat#21126; ThermoFisher), mouse monoclonal antibody against BAX 6A7 (Cat#MA5-14003; RRID: AB_10979735; ThermoFisher), anti-MTCH1 (Cat#PA5100201; RRID: AB_2815731; ThermoFisher), Rabbit polyclonal to mtTFA - Mitochondrial Marker (TFAM) (Cat#ab131607; RRID: AB_11154693; Abcam), β-Actin antibody (Cat#sc-47778; RRID: AB_626632; Santa Cruz), Anti-α-Tubulin antibody, Mouse monoclonal (Cat#T6199; RRID: AB_477583; Sigma), and BID Antibody (Mouse Specific) (Cat#2003S; RRID: AB_10694562; Cell Signaling Technology).

Validation

All antibodies were validated by the supplier.

Eukaryotic cell lines

Policy information about cell lines and Sex and Gender in Research

Cell line source(s)

The experimental models used in the study included the following cell lines and strains:
U2OS wt (Gift from S. Tait, Glasgow; doi.org/10.15252/embj.201899238),
U2OS BAK -/- KO (U2OS BAK KO) (Gift from S. Tait, Glasgow; doi.org/10.15252/embj.201899238),
U2OS BAK -/- BAX -/- DKO (U2OS BAK DKO) (Gift from S. Tait, Glasgow; doi.org/10.15252/embj.201899238),
U2OS BAK -/- KO GFPBAK (U2OS BAK KO GFPBAK) (This manuscript; N/A),
U2OS BAK -/- KO GFPBAK (U2OS BAK KO GFPBAK) MTCH2 KO (This manuscript; N/A),
U2OS BAK -/- KO GFPBAK (U2OS BAK KO GFPBAK) MTCH1 KO (This manuscript; N/A),
U2OS BAK -/- BID -/- KO GFPBAK (U2OS BAK KO GFPBAK) BID KO (This manuscript; N/A),
U2OS BAK -/- BID -/- KO GFPBAK (U2OS BAK KO GFPBAK) BID KO MTCH2 KO (This manuscript; N/A),
U2OS NUP96-mEGFP (Available from CLS cell lines service GmbH; Cat#300174),
Hela wt (ATCC; Cat#CCL2),
Hela Bax-/- BAK -/- DKO (Georg Hacker lab; doi:10.15252/embj.2018100907),
Hela MTCH2 KO (This manuscript; N/A),
MEF wt (Gift from Atan Gross lab; doi:10.1038/s41467-018-07519-w),
MEF MTCH2 KO (Gift from Atan Gross lab; doi:10.1038/s41467-018-07519-w),
MEF MTCH2 KO MTCH2 KI (This manuscript; N/A),
AGS WT (Sigma Aldrich, 89090402-1VL),
AGS MTCH2 KO (This manuscript, N/A),
MEF BAX -/- BAK -/- KO (MEF BAX BAK DKO) (Gift from Villunger lab ;N/A),
AGS BAX -/- BAK -/- KO (AGS BAX/BAK DKO) (doi: 10.1038/s41418-022-01009-9; N/A).

Authentication

Cell lines generated in this study were validated by western blot.

Mycoplasma contamination

All cel lines used in this study were subjected to regular mycoplasma testing using a kit (MycoStrip,Invivogen).

Commonly misidentified lines
(See ICLAC register)

none of the cell lines used are commonly misidentified lines.

Animals and other research organisms

Policy information about studies involving animals; ARRIVE guidelines recommended for reporting animal research, and Sex and Gender in Research

Laboratory animals	NA
Wild animals	NA
Reporting on sex	NA
Field-collected samples	NA
Ethics oversight	NA

Note that full information on the approval of the study protocol must also be provided in the manuscript.

Plants

Seed stocks	NA
Novel plant genotypes	NA
Authentication	NA
