# Supplementary figures and images for: MTCH2 promotes BAX and BAK self-assembly and apoptotic pore growth
Source: Nat Struct Mol Biol. 2026 Apr 29;33(5):824–37. doi: 10.1038/s41594-026-01805-8 (PMC13186707; doi:10.1038/s41594-026-01805-8)

Figure 1C

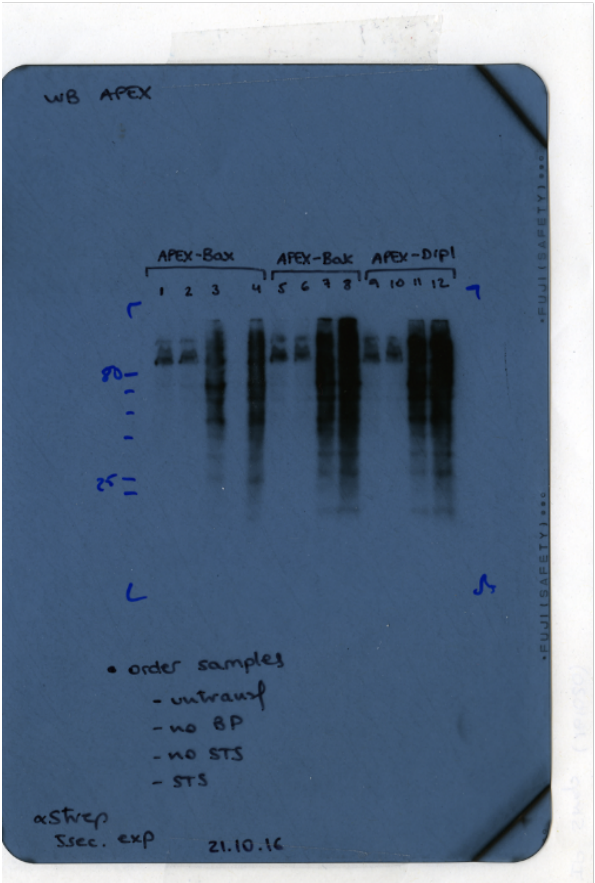

| APEX2 | BAX |   |   |   | BAK |   |   |   | DRP1 |   |   |   |
|-------|-----|---|---|---|-----|---|---|---|------|---|---|---|
| BP    | +   | - | + | + | +   | - | + | + | +    | - | + | + |
| STS   | +   | + | - | + | +   | + | - | + | +    | + | - | + |

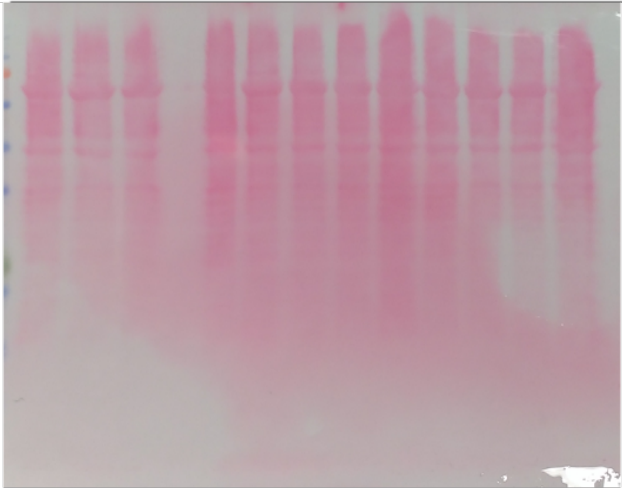

Supplement: Supplementary file 18 — Unprocessed WB gels. [file 41594_2026_1805_MOESM18_ESM.pdf]

**Figure 2F**

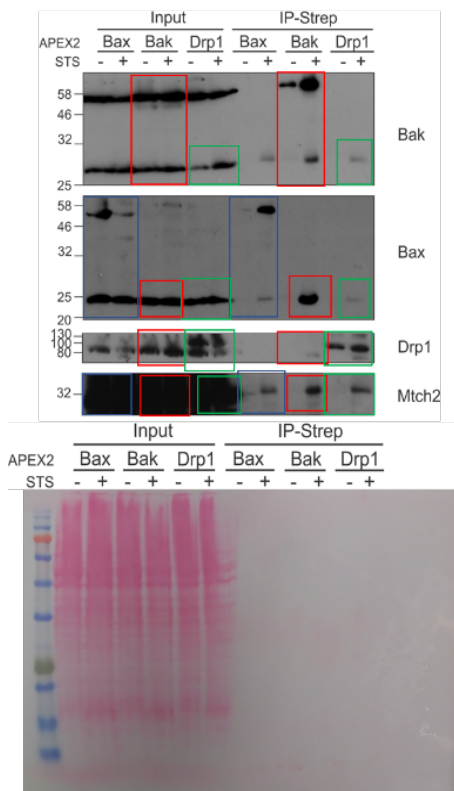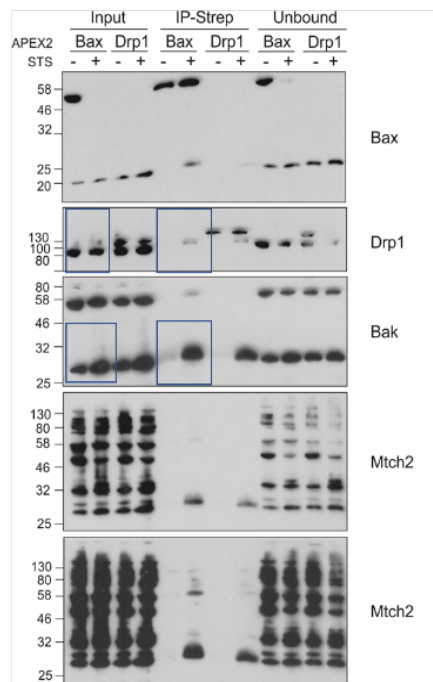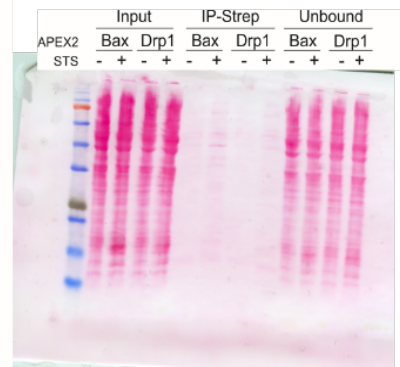

Supplement: Supplementary file 19 — Unprocessed WB gels. [file 41594_2026_1805_MOESM19_ESM.pdf]

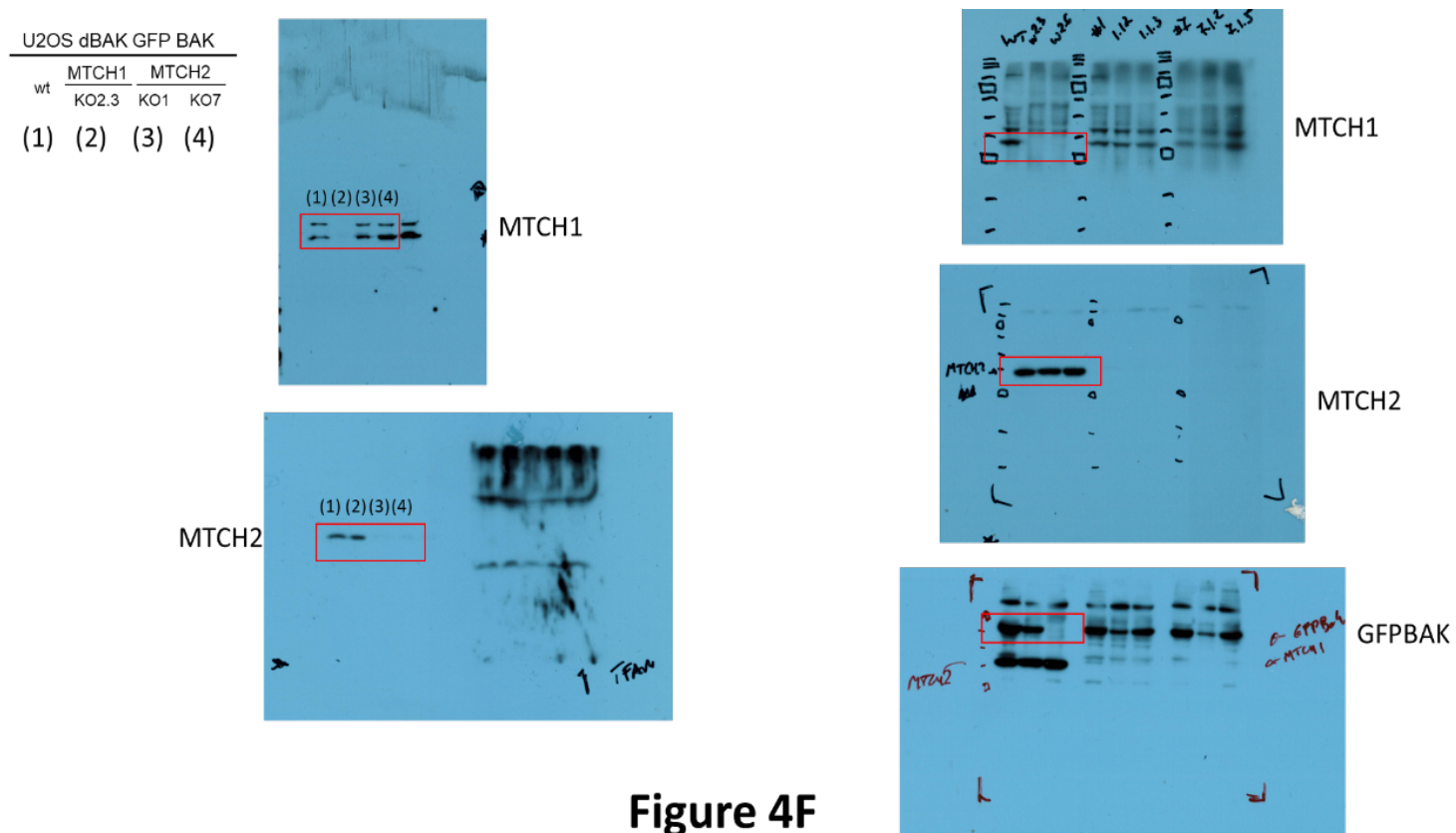

**Figure 4F**

Supplement: Supplementary file 20 — Unprocessed WB gels. [file 41594_2026_1805_MOESM20_ESM.pdf]

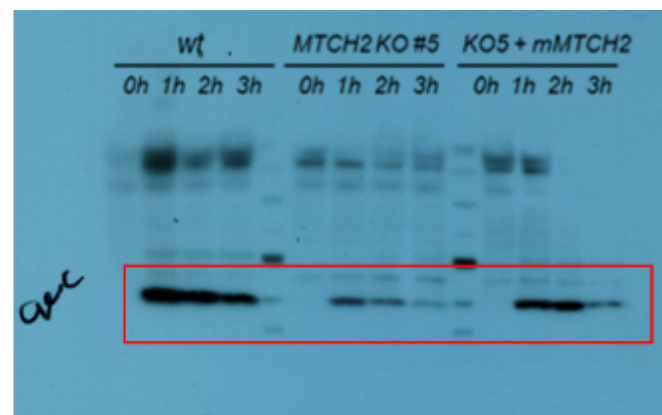

Cyt c

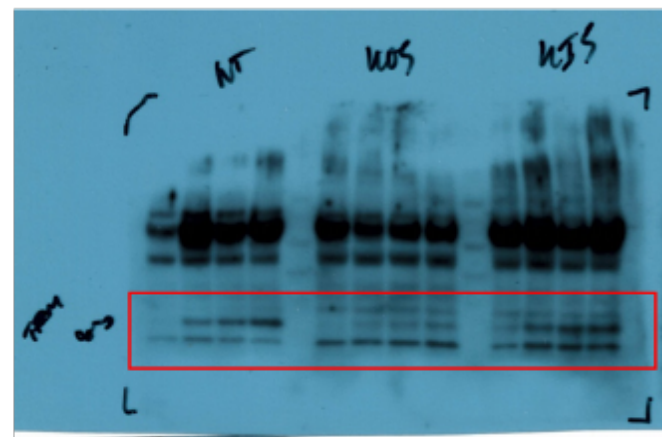

TFAM

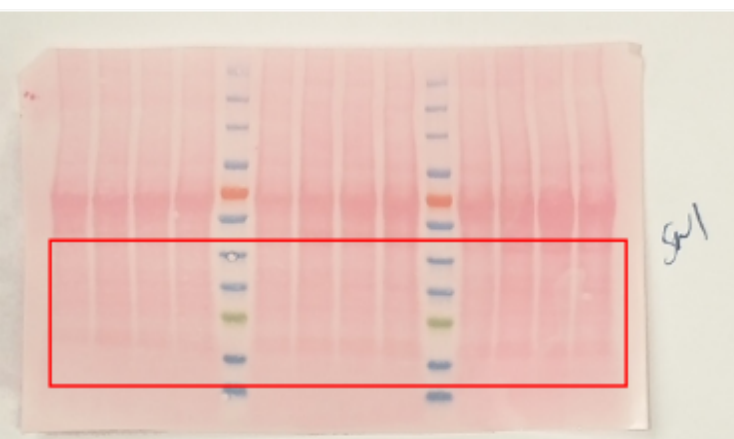

Figure 6F

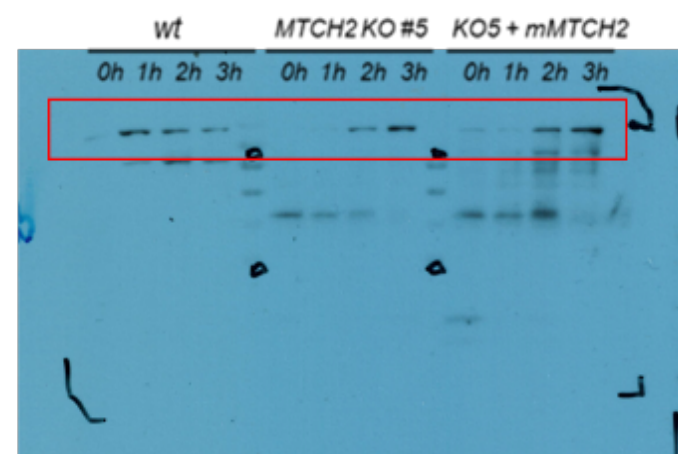

TBK1-P

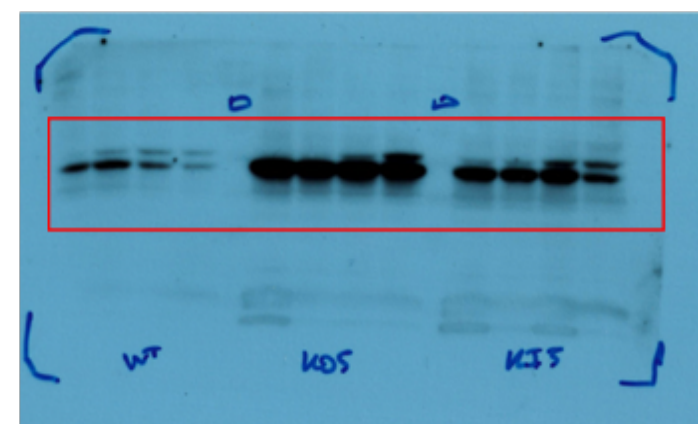

STING

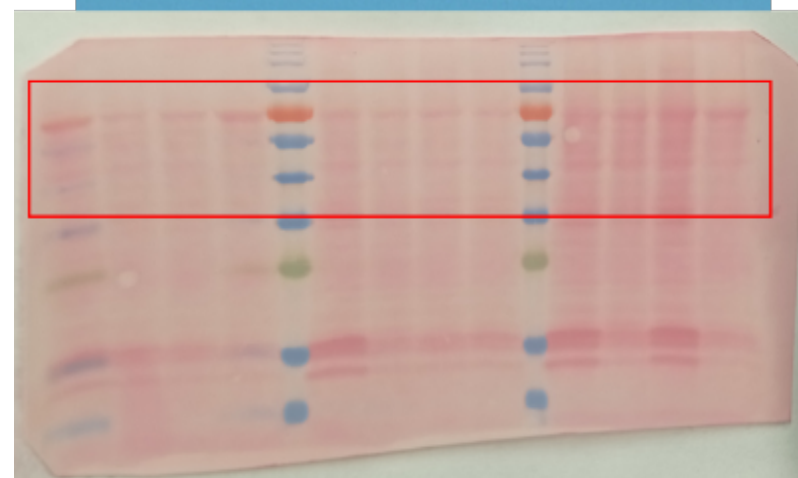

Figure 6G

Supplement: Supplementary file 21 — Unprocessed WB gels. [file 41594_2026_1805_MOESM21_ESM.pdf]

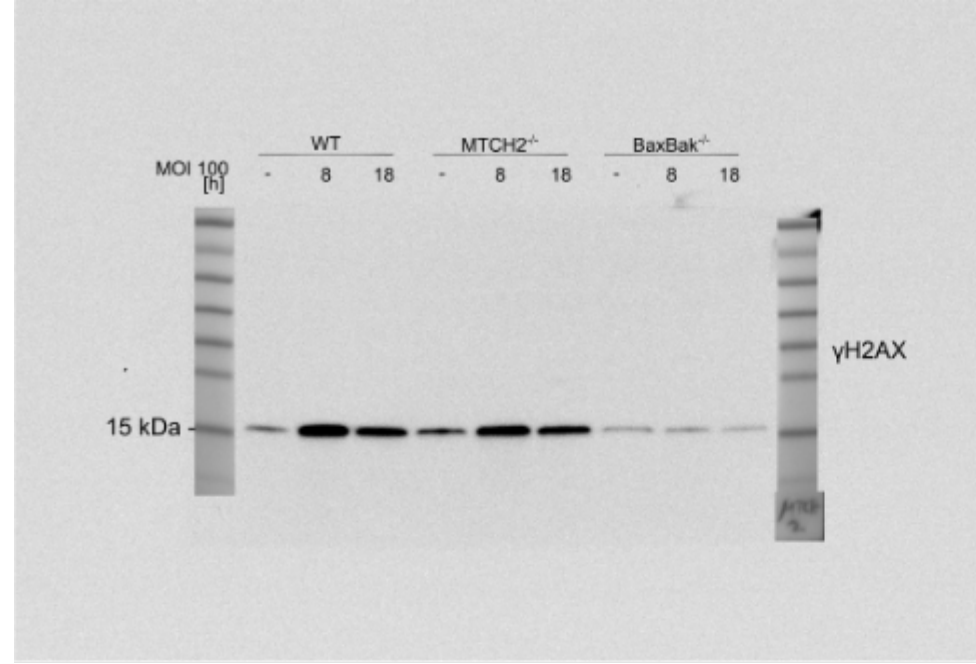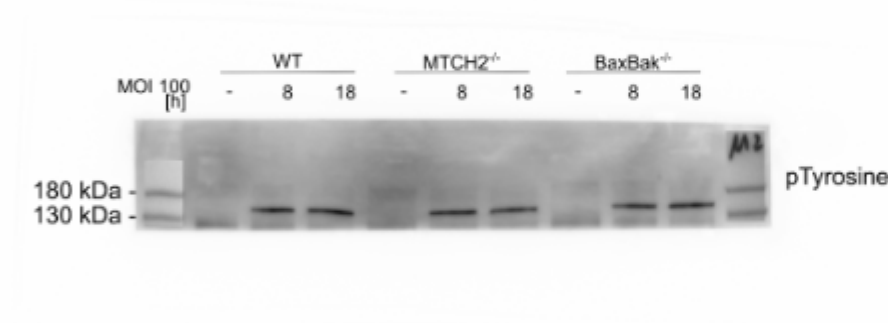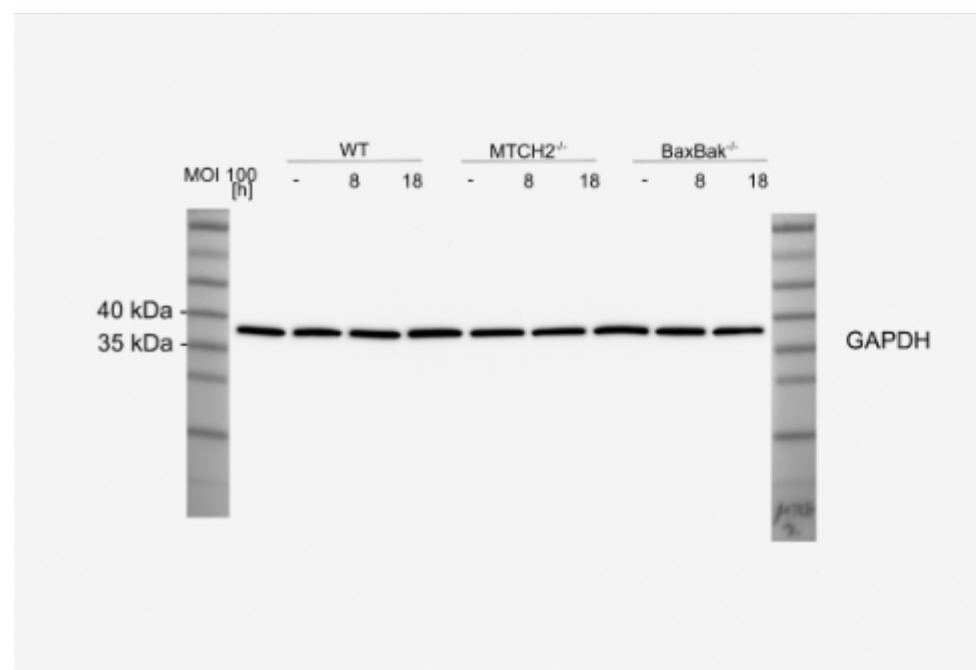

Figure 7D

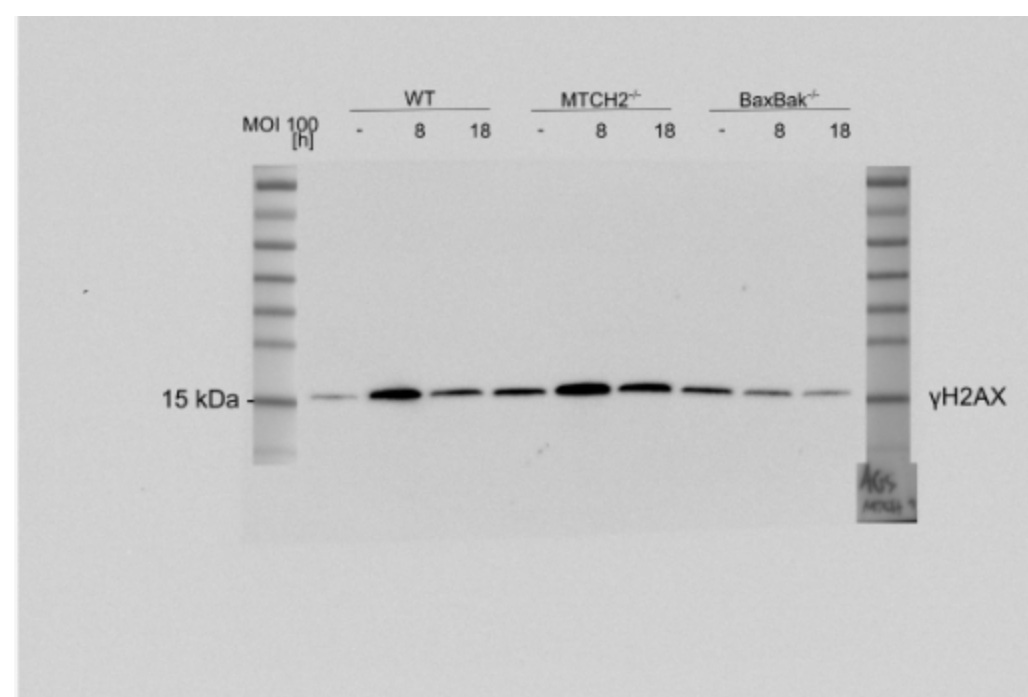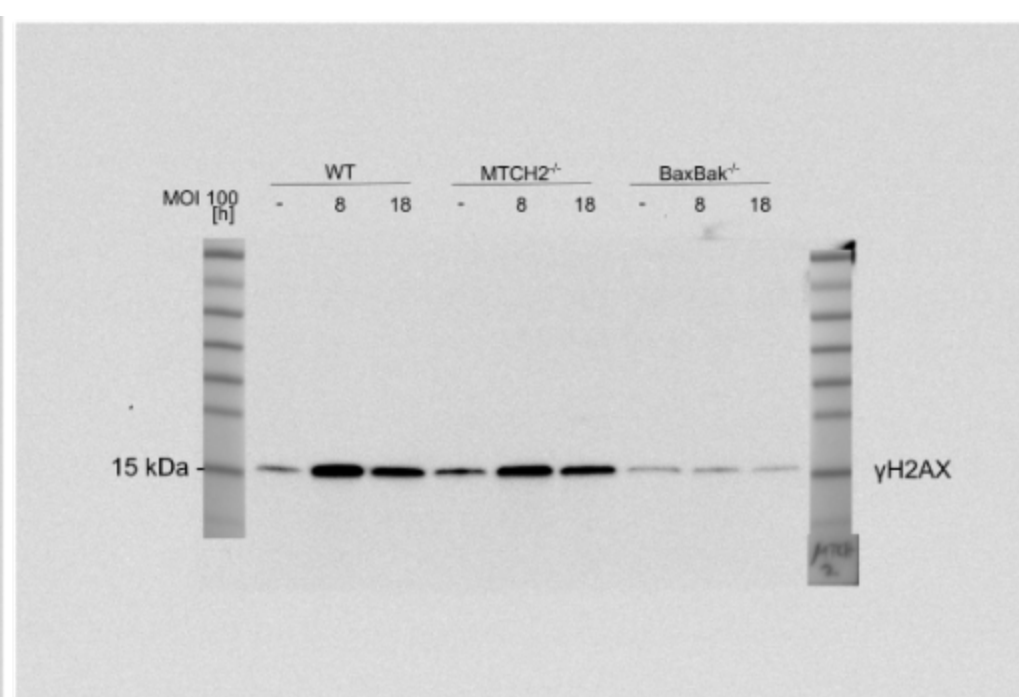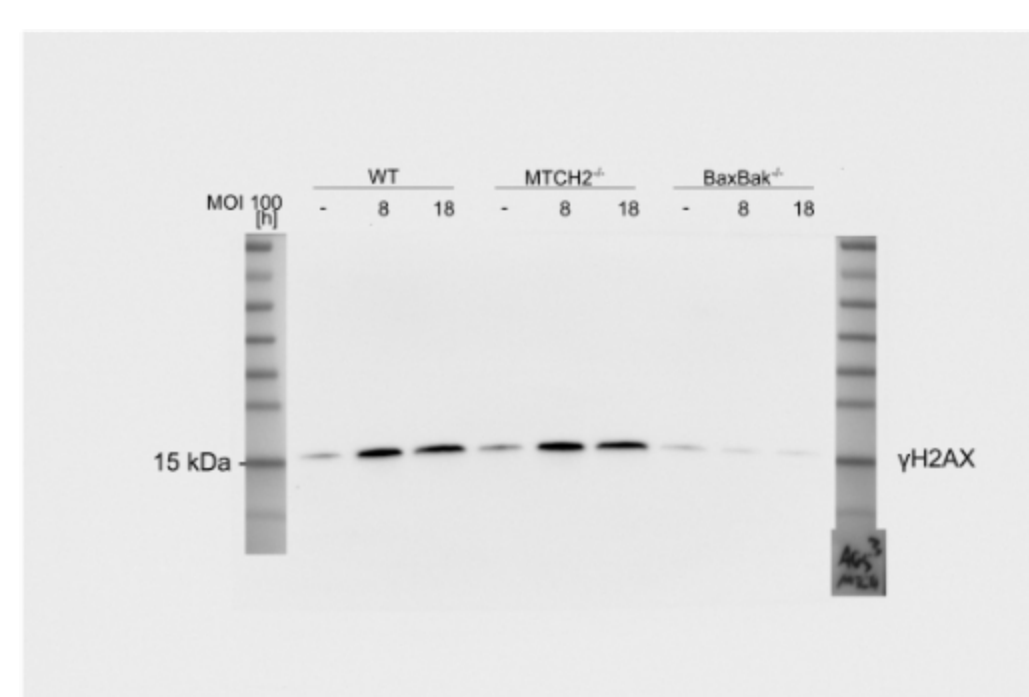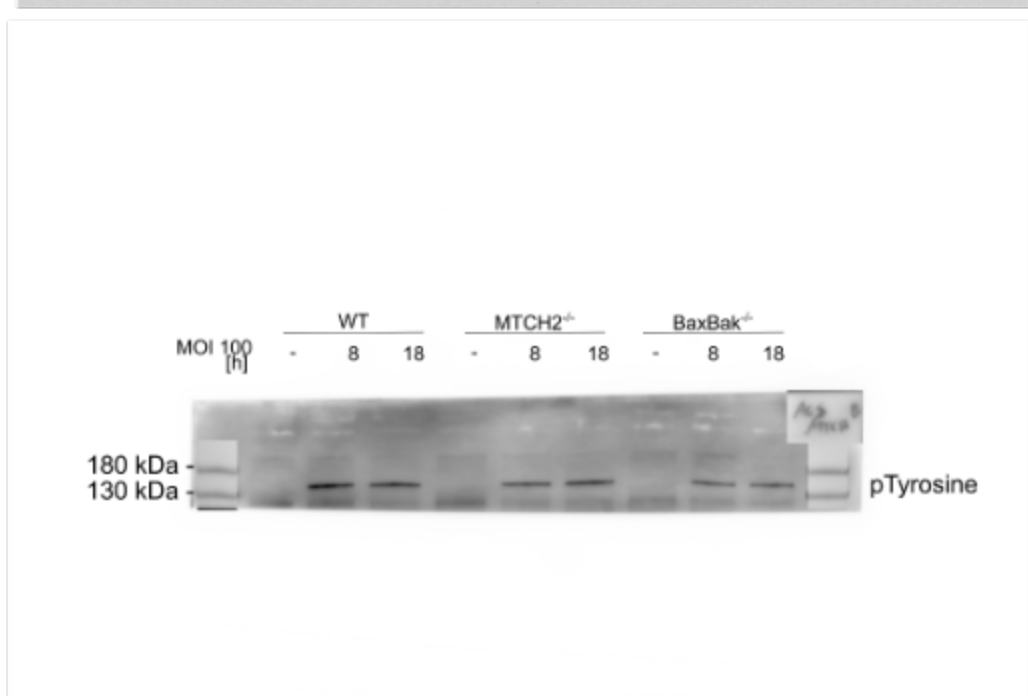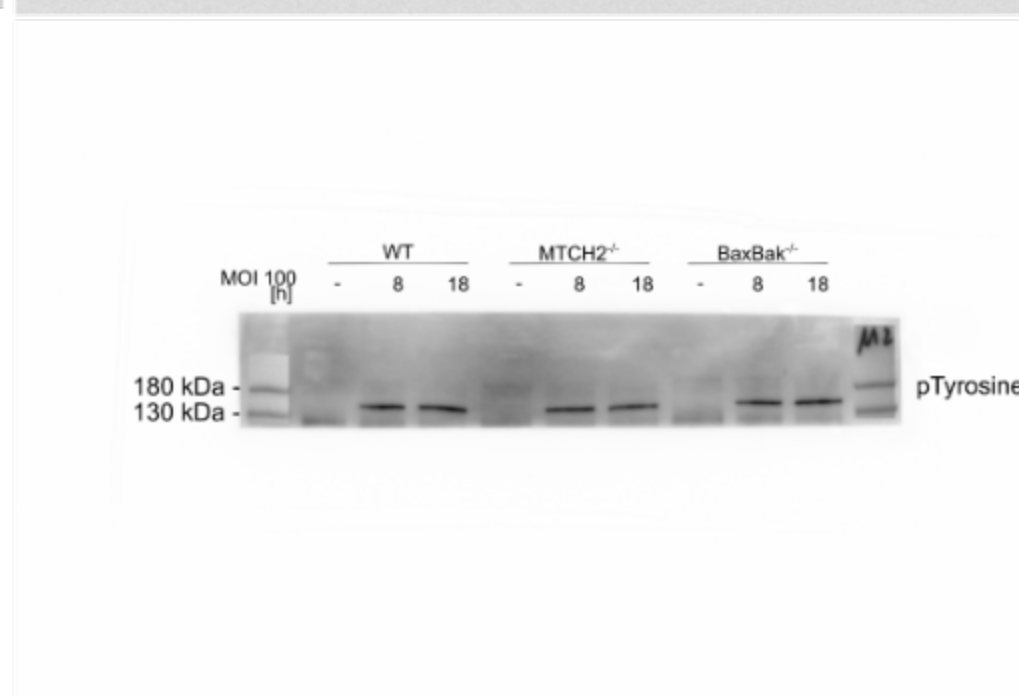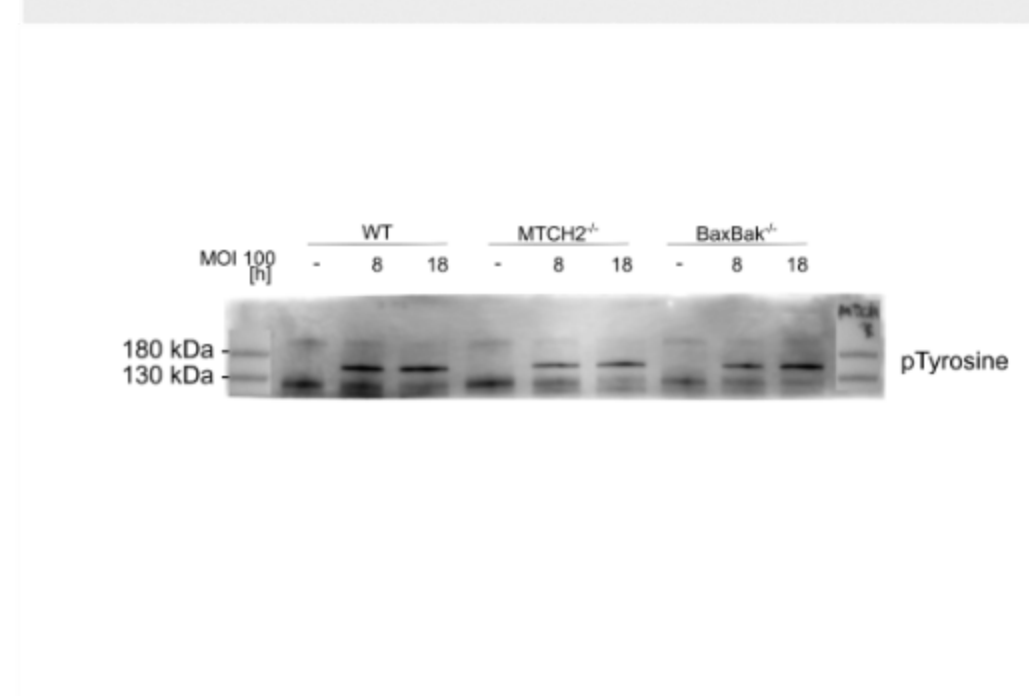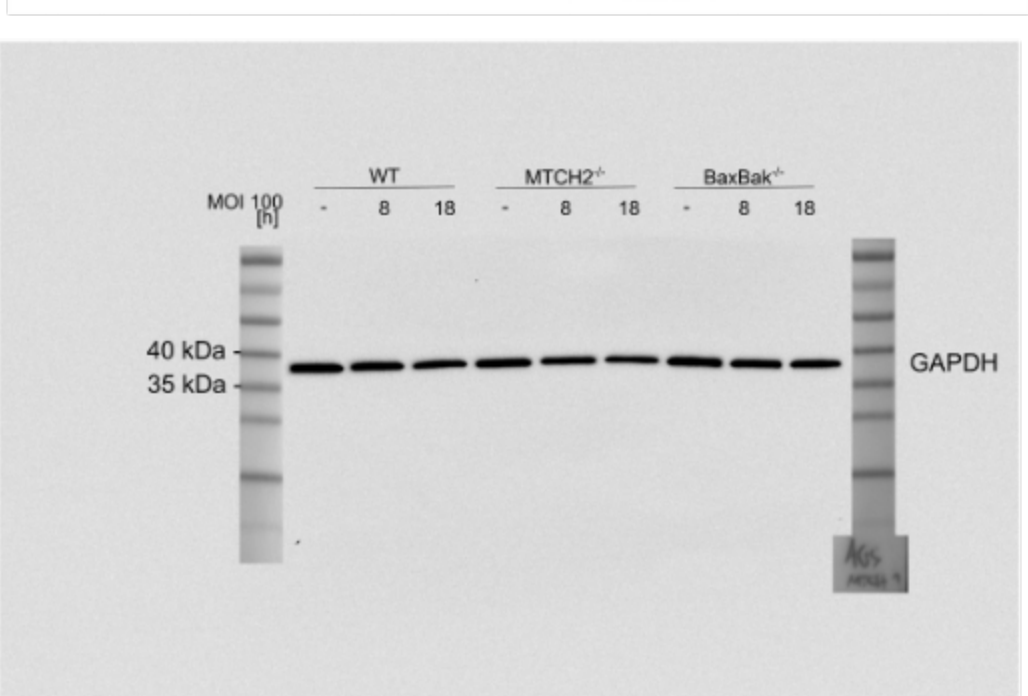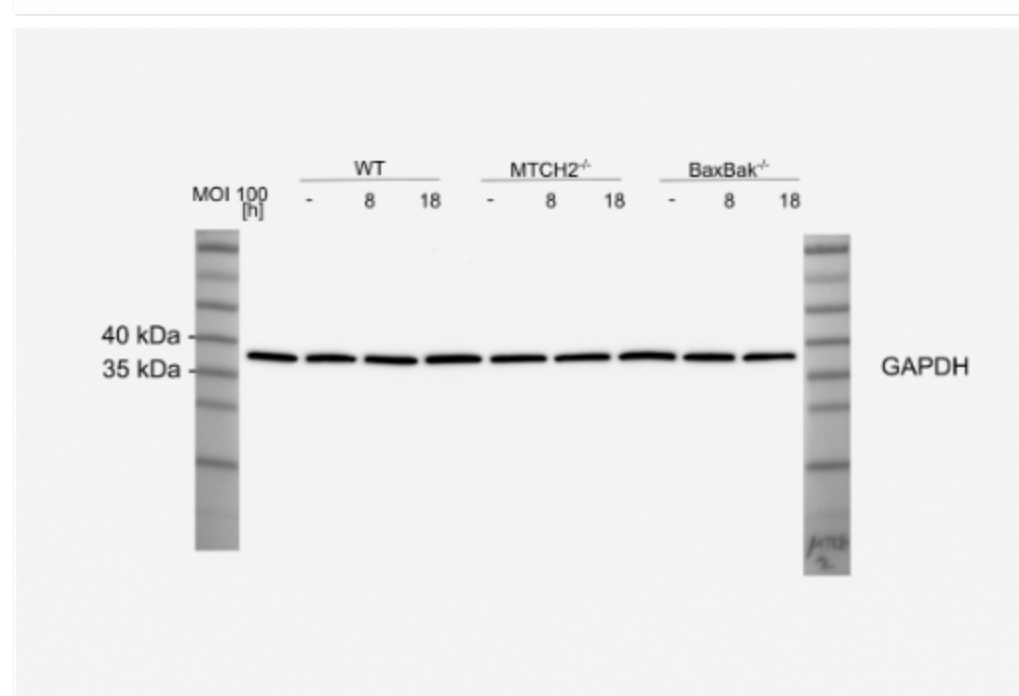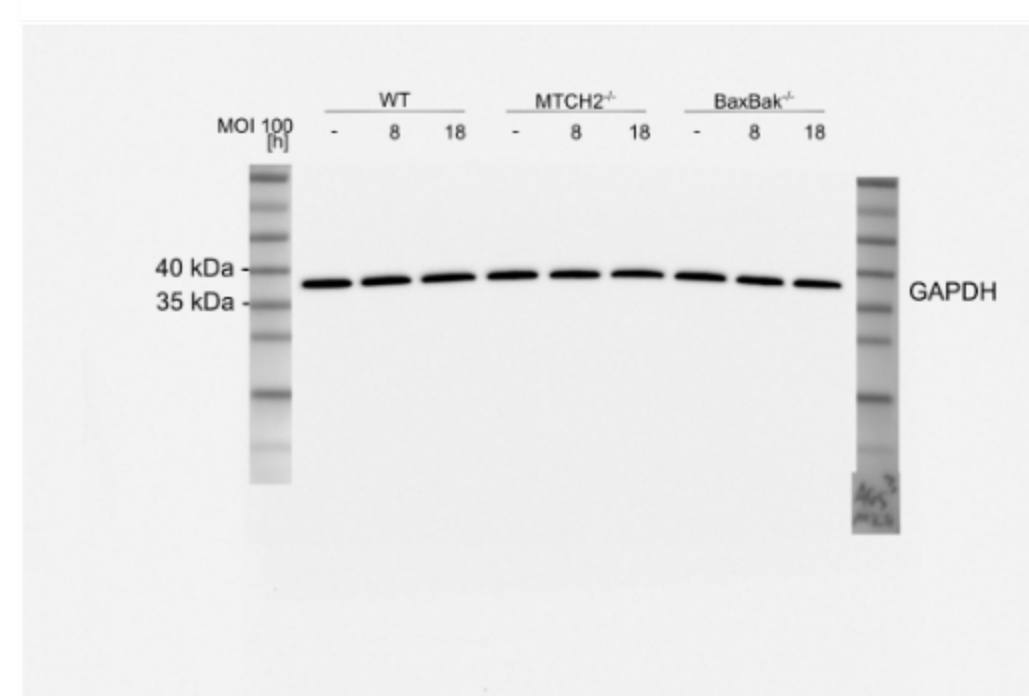

Figure 7E

Supplement: Supplementary file 22 — Unprocessed WB gels. [file 41594_2026_1805_MOESM22_ESM.pdf]
